# Toward maximum energy density enabled by anode‐free lithium metal batteries: Recent progress and perspective

**DOI:** 10.1002/EXP.20210255

**Published:** 2023-09-26

**Authors:** Cheol‐Young Park, Jinuk Kim, Won‐Gwang Lim, Jinwoo Lee

**Affiliations:** ^1^ Chemical and Biomolecular Engineering Korea Advanced Institute of Science and Technology (KAIST) Daejeon Republic of Korea; ^2^ Present address: Energy and Environment Directorate Pacific Northwest National Laboratory (PNNL), 902 Battelle Boulevard Richland 99354 Washington USA

**Keywords:** advanced electrolytes, anode‐free lithium metal batteries, current collectors

## Abstract

Owing to the emergenceof energy storage and electric vehicles, the desire for safe high‐energy‐density energy storage devices has increased research interest in anode‐free lithium metal batteries (AFLMBs). Unlike general lithium metal batteries (LMBs), in which excess Li exists to compensate for the irreversible loss of Li, only the current collector is employed as an anode and paired with a lithiated cathode in the fabrication of AFLMBs. Owing to their unique cell configuration, AFLMBs have attractive characteristics, including the highest energy density, safety, and cost‐effectiveness. However, developing AFLMBs with extended cyclability remains an issue for practical applications because the high reactivity of Li with limited inventory causes severely low Coulombic efficiency (CE), poor cyclability, and dendrite growth. To address these issues, tremendous effort has been devoted to stabilizing Li metal anodes for AFLMBs. In this review, the importance and challenges of AFLMBs are highlighted. Then, diverse strategies, such as current collectors modification, advanced electrolytes, cathode engineering, and operation protocols are thoroughly reviewed. Finally, a future perspective on the strategy is provided for insight into the basis of future research. It is hoped that this review provides a comprehensive understanding by reviewing previous research and arousing more interest in this field.

## Graphical abstract

To comprehend the anode‐free lithium metal batteries, it is gone into detail in this review about current collector modification, electrolyte optimization, cathode engineering, and regulation of cycling parameters.

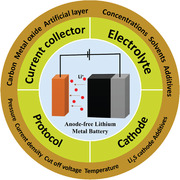

## 1 INTRODUCTION

**SCHEME 1:**
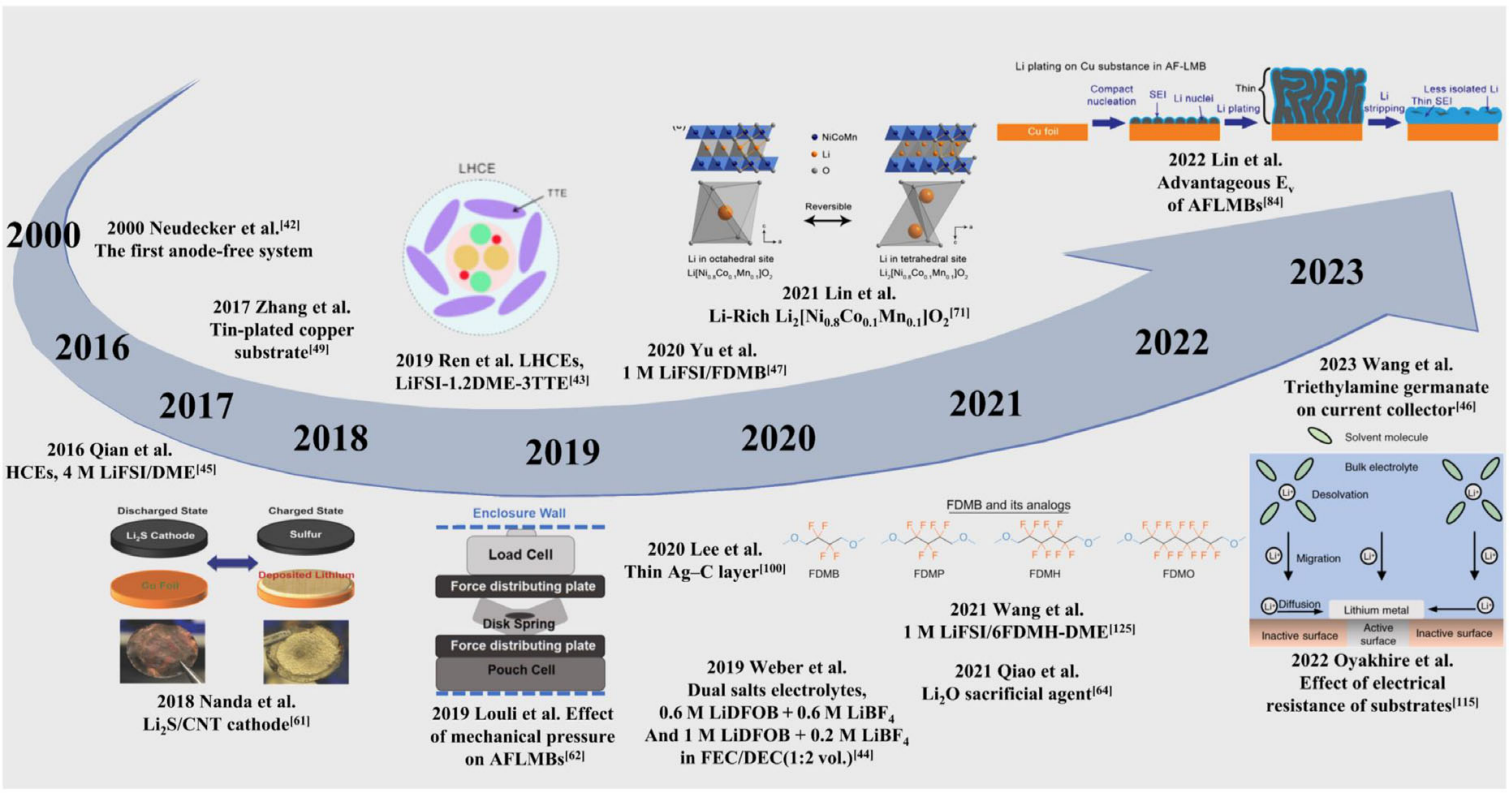
Historical timeline of anode‐free lithium metal batteries (AFLMBs). Reproduced with permission.^[^ ^43^, ^61^, ^67^, ^71^, ^84^, ^115^, ^125^ ^]^ Copyright, Wiley‐VCH, The Electrochemical Society and Elsevier.

Since the first commercialization of lithium‐ion batteries (LIBs) by Sony Corp. in 1991, LIBs have been successfully used in applications ranging from small portable devices to grid energy storage systems.^[^ ^1^, ^2^ ^]^ In the 21st century, global environmental issues have driven the development of electric vehicles (EVs) and renewable energy, which require greater energy storage density. However, state‐of‐the‐art LIBs have almost reached the theoretical limit of energy density (≈300 Wh kg^−1^) because of the low theoretical capacity of intercalation‐type electrode materials (e.g. graphite and lithium metal oxides).^[^ ^3^, ^4^, ^5^ ^]^ Therefore, next‐generation anodes such as alkali metals (Li, Na,^[^ ^6^, ^7^, ^8^, ^9^, ^10^ ^]^ K,^[^ ^11^, ^12^, ^13^, ^14^ ^]^), alkaline earth metals (Mg,^[^ ^15^, ^16^, ^17^, ^18^, ^19^ ^]^ Ca^[^ ^20^, ^21^, ^22^, ^23^, ^24^ ^]^), and multivalent metals (Zn,^[^ ^25^, ^26^, ^27^, ^28^, ^29^ ^]^ Al^[^ ^30^, ^31^ ^]^) are in the spotlight to go beyond LIBs. Among them, Li metal has gained the most attention as a next‐generation anode material to overcome the theoretical limitations of intercalation‐type anodes.^[^ ^4^, ^32^ ^]^ Li metal has very promising characteristics for high energy density, namely the lowest reduction potential (−3.04 V vs standard hydrogen electrode, SHE), high theoretical capacity (3860 mA h g^−1^), and being lightweight (0.53 g cm^−3^). However, thick Li metal anodes (>250 μm) are frequently used in lithium metal batteries (LMBs)^[^ ^33^, ^34^ ^]^ to compensate for the irreversible loss of electrically isolated dead Li^[^ ^35^, ^36^ ^]^ and solid‐electrolyte interphase (SEI) layer generation.^[^ ^37^, ^38^, ^39^ ^]^ This configuration extends the cyclability of the cells but significantly lowers the energy density, which is often lower than that of LIBs.^[^ ^4^, ^39^ ^]^ To overcome the above issue, thin Li metal (20–50 μm) electrodes have been employed. Recently, an anode composed of a sole current collector without Li metal was used by pairing it with a lithiated cathode, where the negative‐to‐positive (N/P) ratio is 0. The batteries with this simple configuration are called anode‐free lithium metal batteries (AFLMBs), which were first developed by Neudecker et al. in 2000 (Scheme 1).^[^ ^40^ ^]^ Maximum energy density can be achieved because of the ideal environment.^[^ ^41^, ^42^ ^]^ Despite the considerable advantages of the anode‐free configuration, the cycle stability of AFLMBs is too poor because the absence of a Li reservoir in the anode causes rapid degradation of the cycle.^[^ ^43^, ^44^ ^]^ As shown in Scheme 1, AFLMBs have received significant attention since a breakthrough by Qian et al. in 2016,^[^ ^45^ ^]^ who initiated an anode‐free boom by proving the feasibility of AFLMBs. Even now, more and more advanced strategies are being conducted to extend the cycle stability of AFLMBs.^[^ ^46^ ^]^ Various efforts have been made to enhance the cyclability of AFLMBs.^[^ ^44^, ^47^ ^]^ In this review, we categorize the various strategies for high‐performance AFLMBs into three types. The first is the rational design of current collectors to enhance the reversibility of Li metal. The construction of elaborate 3D structures,^[^ ^48^ ^]^ diverse lithiophilic sites,^[^ ^49^ ^]^ artificial layers on current collectors,^[^ ^50^, ^51^ ^]^ and carbon hosts^[^ ^52^ ^]^ have been explored to reduce the nucleation energy barrier, lead to uniform deposition of Li, and suppress dendrites. The next one is electrolyte engineering. The SEI layer formed by electrolyte decomposition has the greatest effect on the reversibility of Li metal.^[^ ^44^, ^47^, ^53^, ^54^, ^55^, ^56^, ^57^, ^58^ ^]^ Various studies have fabricated high‐quality SEI layers, including by adjusting salt concentration,^[^ ^45^ ^]^ changing solvent characteristics,^[^ ^47^, ^59^ ^]^ and introducing additives.^[^ ^54^, ^60^ ^]^ Then, we introduce a strategy through cathode engineering. This is achieved through the unique properties of the Li_2_S cathode system^[^ ^61^, ^62^, ^63^ ^]^ or the introduction of lithiated additives into the cathode.^[^ ^64^, ^65^ ^]^ The last category is operating protocols. Various parameters such as temperature, external pressure, current density, and cut‐off voltages dramatically affect the cycle stability.^[^ ^66^, ^67^, ^68^ ^]^

**FIGURE 1:**
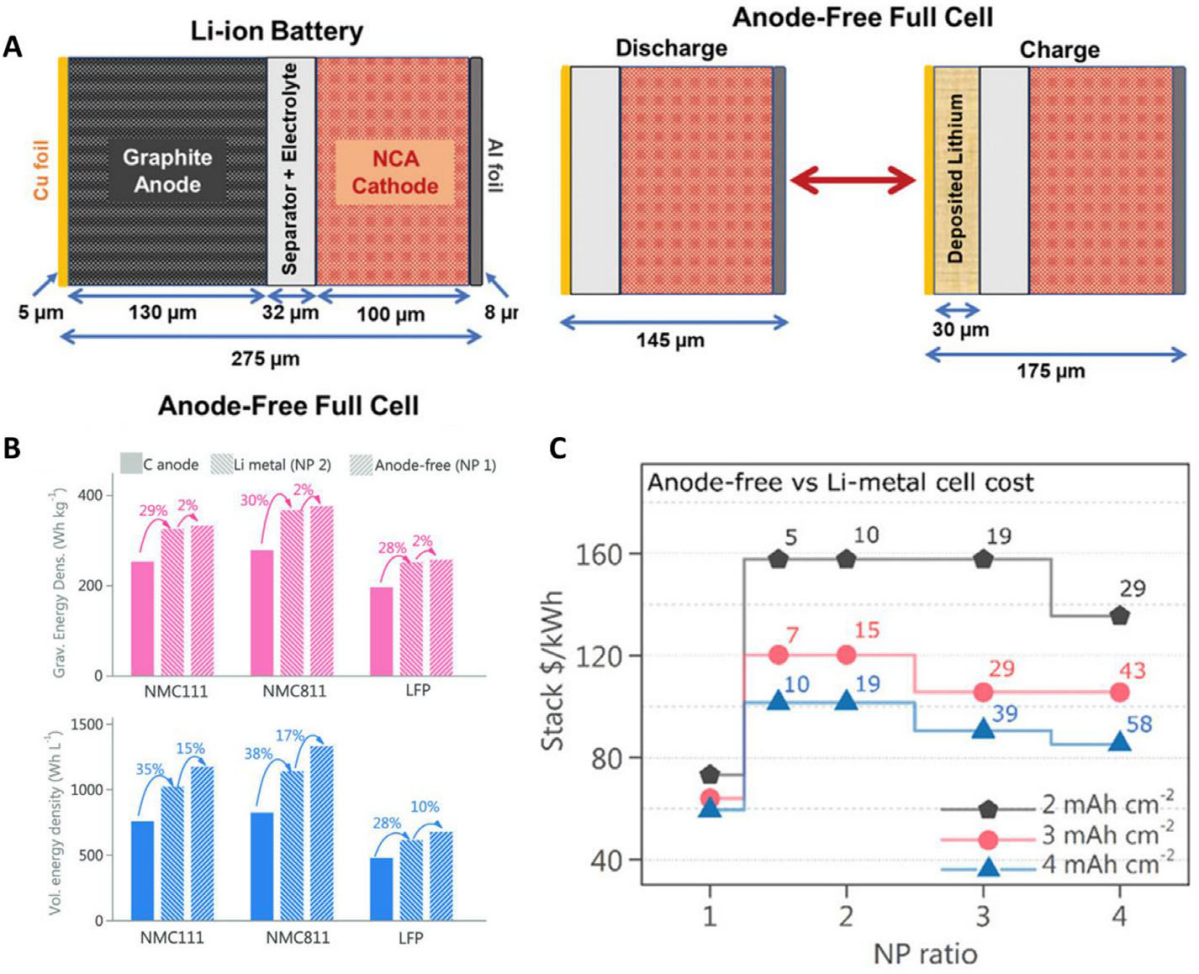
Advantages of anode‐free lithium metal batteries (AFLMBs). (A) Schematic illustration of Li‐ion batteries and anode‐free full cell. Reproduced with permission.^[^ ^61^ ^]^ Copyright 2021, Wiley‐VCH. (B,C) Volumetric and gravimetric energy density of AFLMBs with different cathodes and cost of stack $/kWh according to capacity and N/P ratio. Reproduced with permission.^[^ ^70^ ^]^ Copyright 2021, Wiley‐VCH.

The initial cell configuration of AFLMBs is the same as that of general LMBs, except that the anode is composed of sole current collectors without Li metal (Figure 1A). Therefore, lithiated cathode materials are necessary for AFLMBs. Generally, intercalation materials such as lithium cobalt nickel manganese oxide (NMC),^[^ ^44^ ^]^ lithium iron phosphate (LFP),^[^ ^45^, ^69^ ^]^ lithium nickel cobalt aluminum oxide (NCA), or conversion‐type (e.g. Li_2_S) cathodes^[^ ^61^, ^63^ ^]^ are utilized as cathodes for AFLMBs. During the initial charging process, Li‐ions are delithiated from the cathode and reduced on the surface of the current collectors in the anode. The subsequent discharge and charge processes progressed similarly to the LMBs.

### 1.1 Advantageous features of AFLMBs

#### 1.1.1 Volumetric energy density (*E_v_*)

As shown in Figure 1B there are tremendous effects on full cells by substitution of graphite to Li metal anode (3 mA h cm^−2^, N/P ratio = 1) on both specific gravimetric and volumetric energy density (*E_g_*, *E_v_*). Furthermore, the *E_g_* and *E_v_* of AFLMBs are the maximum values that can be achieved by regulating cell configurations. In the case of *E_g_*, only a slight improvement in *E_g_* (2%) of AFLMBs over LMBs is achieved because Li metal is only a small portion of the total mass of the cell compared to other components (mainly current collector and electrolytes) due to the lightweight nature and high specific capacity of Li.^[^ ^70^ ^]^ However, anode‐free configurations have a huge impact on *E_v_* over LMBs even under a fully charged state. Compared to LMBs using very thin Li metal, a 10−17% *E_v_* improvement is observed depending on the cathode material. The highest *E_v_* of AFLMBs must be efficient when the battery needs to fit into a fixed volume, such as in EVs.

#### 1.1.2 Low nucleation barrier and high reversibility of Li

Unlike LMBs, Li nucleates directly on the surface of the current collector and subsequently grows during the initial charge process for AFLMBs. Owing to the higher nucleation kinetics of Li on Cu than on Li metal, uniform Li nucleates, and compact Li metal is deposited.^[^ ^71^ ^]^ The non‐uniform and porous morphology of Li plated on Li metal is detrimental to cyclability, whereas uniform and dense Li metal growth on Cu CCs reduces the side reactions of Li and the electrolyte, resulting in high CE.

#### 1.1.3 Cost

To attain a practical energy density (>350 W h kg^−1^), the cathode capacity should be above 3−4 mA h cm^−2^ and a thin Li anode (N/P ratio < 2) is a prerequisite.^[^ ^4^, ^72^ ^]^ To meet these criteria, a Li metal anode of 15−20 μm should be employed. In general, the calendaring process to produce ultrathin Li foil significantly increases production costs (Li < 20 μm—$13 m^−2^, Li 20–50 μm—$9.6 m^−2^, Li > 50 μm—$8 m^−2^).^[^ ^70^ ^]^ Figure 1C represents the stack cost per energy estimation of the cells. Interestingly, LMBs with thinner Li anodes (2 mA h cm^−2^) are more expensive than those with thicker Li anodes (4 mA h cm^−2^), although less Li is utilized because of the high cost of the Li calendaring process. Meanwhile, it is possible to dramatically reduce the production cost while increasing the energy density, as AFLMBs do not require excess Li. In addition to excluding the price of the Li processing, less labor is required, and the convenience of the process is increased because highly reactive Li metal is excluded from all manufacturing processes. Furthermore, there is no need to make new investments in manufacturing facilities because of their compatibility with the LIBs fabrication process.^[^ ^70^, ^73^ ^]^

#### 1.1.4 Measuring tool

AFLMBs are appropriate for measuring the reversibility of Li. In the case of LMBs, the cycle stability is often exaggerated by thick Li metal, hiding the degradation of active Li until the depletion of the Li reservoir.^[^ ^74^, ^75^ ^]^ Therefore, LMBs are not suitable systems for measuring the true reversibility of Li. For AFLMBs, however, the Coulombic efficiency (CE) directly presents the consumption of Li in every cycle because the inactivated Li is not compensated. Therefore, an anode‐free configuration was used to determine the degree of reversibility of Li.^[^ ^68^, ^74^ ^]^

### 1.2 Challenges of AFLMBs

#### 1.2.1 Low reversibility of Li

The absence of excess Li metal brings not only huge advantages but also causes rapid capacity decay of AFLMBs within a few cycles. Owing to the high reactivity of Li metal, active Li is continuously consumed by the reaction with the electrolytes and the formation of dead Li.^[^ ^35^, ^76^, ^77^, ^78^, ^79^ ^]^ Without excess Li metal, CEs are directly related to the reversibility of the Li stripping/plating process. The correlation between the CE and cycle life is shown in Table 1.^[^ ^42^ ^]^ High CE (>99.978%) is required for AFLMBs to practically compete with commercial LIBs (>500 cycles), but this has not yet been achieved. Even when CE is highly improved to 99.8%, the cell only exhibits 80% capacity retention after only 100 cycles, which is far below the commercialization. Therefore, even if a high initial energy density is achieved through AFLMBs, it drops rapidly within several cycles.

**TABLE 1:** Coulombic efficiency for 80% retention of initial capacity. Reproduced with permission.^[^ ^45^ ^]^ Copyright 2016, Wiley‐VCH.

CE [%]	Cycles to maintain a capacity > 80% of the initial value
99	22
99.1	25
99.7	74
99.8	112
99.9	223
99.99	2231

#### 1.2.2 Surface impurities of the current collector

In the case of AFLMBs, the direct deposition of Li on the surface of the current collector is inevitable. However, impurities or a non‐uniform native oxide layer on the surface of the current collector consume active Li and increase the Li nucleation barrier, resulting in adverse effects on the morphology of the initial and subsequent growth of Li.^[^ ^80^ ^]^ Furthermore, the uneven surface properties of the current collector can induce preferential growth of Li, causing dendrite formation. Therefore, it is important to maintain the surface of the current collector by removing impurities and storing them to prevent contamination to improve the reversibility of Li.

#### 1.2.3 Continuous consumption of Li by SEI layer and dead Li

Li is a very strong reducing agent because of the lowest redox potential (−3.04 V vs SHE). Thus, Li reacts intensely with the electrolytes, forming the SEI layer, which passivates the Li surface to prevent further consumption of Li and electrolytes.^[^ ^51^ ^]^ However, the SEI layer is usually cracked owing to the large volume change during the charge/discharge process,^[^ ^53^, ^81^ ^]^ so that fresh Li is inevitably exposed to the electrolytes, leading to the repetitive loss of Li and electrolytes. A thick and non‐uniform SEI layer formed by the repeated formation of SEI interferes with Li‐ion conduction and induces uneven and dendritic Li deposition. This unfavorable morphology of Li is susceptible to the formation of electrically isolated dead Li,^[^ ^35^, ^77^, ^78^, ^79^ ^]^ which accelerates active Li loss.^[^ ^82^ ^]^

#### 1.2.4 Galvanic corrosion

In addition to Li loss from direct contact between the electrolyte and Li, galvanic corrosion of Li occurs during the rest period because of spontaneous electron flow due to the difference in the redox potential.^[^ ^83^ ^]^ During the initial charge process of AFLMBs, Cu substrates are easily exposed to electrolytes. Because the porous, organic‐rich SEI formed on the surface of Cu current collectors cannot prevent the entire surface of Cu, the exposed Cu surface becomes a pathway for electron transfer. The electrons from the oxidation of Li are transported to the electrolyte through the exposed Cu, causing the loss of Li with a pit shape and decomposition of the electrolyte simultaneously. Eventually, dendrites were induced during the charging process. Galvanic corrosion, which can significantly reduce the reversibility of Li during the resting period, has not yet been taken seriously; however, considering the actual operation of AFLMBs, much attention is needed.

Despite the complicated challenges to overcome, the potential benefits of AFLMBs must be apparent. With tremendous lessons learned from both LIBs and LMBs, there have been rapid developments in extending the limited cyclability of AFLMBs. In this review, we discuss current collector modification, electrolyte optimization, cathode engineering, and regulation of cycling parameters in detail to provide an understanding of its impacts and obtain insights. Finally, we provide perspectives on various strategies emphasizing the practicality of AFLMBs.

## 2 CURRENT COLLECTOR ENGINEERING

### 2.1 The significance of current collector modification

**FIGURE 2:**
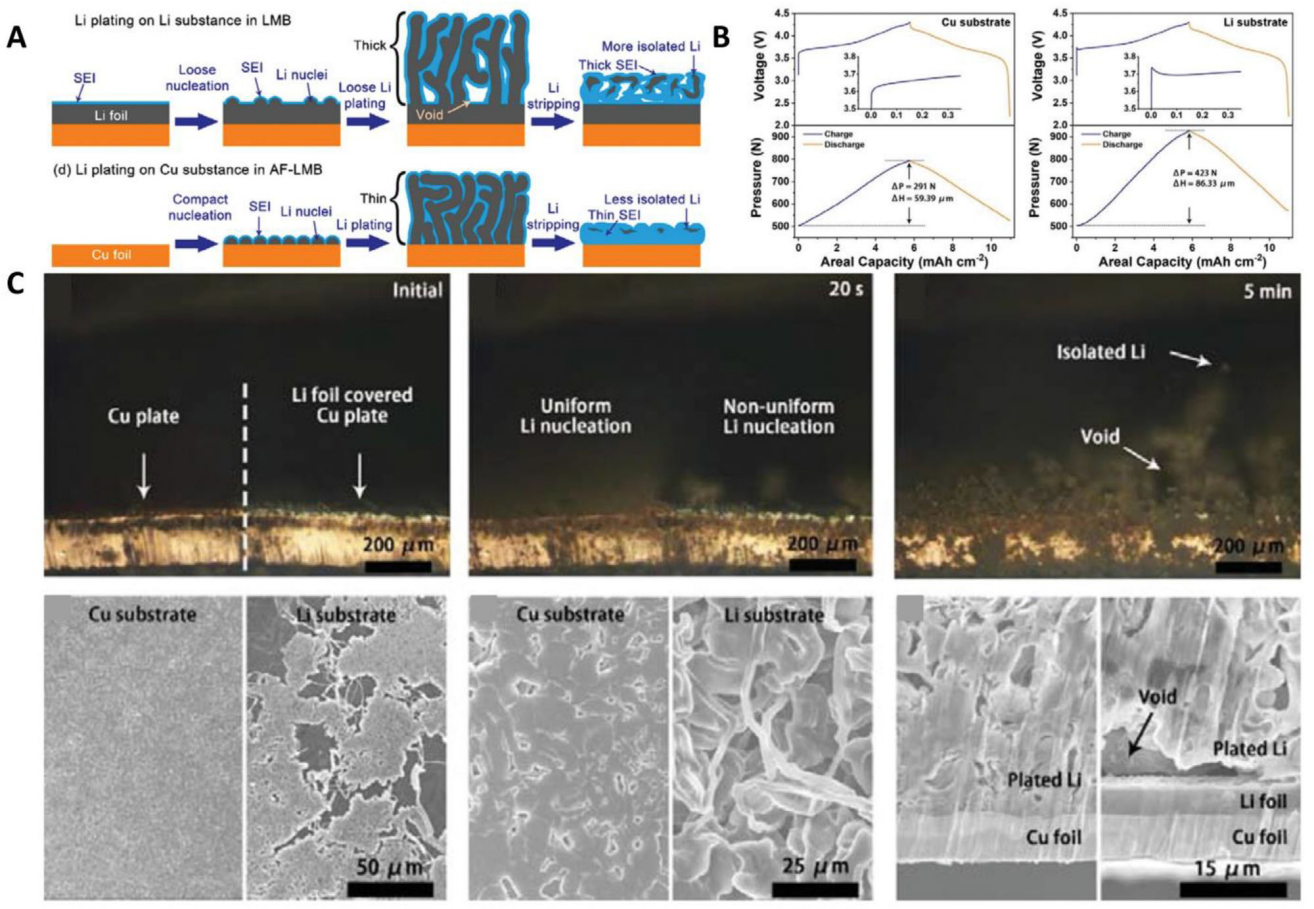
Advantages of Li metal plating on Cu versus Li. (A) Schematic illustration of Li metal plating on Li and Cu. (B) Voltage profiles and the corresponding pressure profiles of Cu||NCM811 and Li||NCM811. (C) Morphologies of Li plating on Cu and Li, observed by optical microscope (OM) and scanning electron microscope (SEM). Reproduced with permission.^[^ ^84^ ^]^ Copyright 2022, Wiley‐VCH.

As stated above, the behavior and morphology of Li plating are significantly different from those of LMBs (Figure 2A).^[^ ^84^ ^]^ Recently, Lin et al. measured the pressure and thickness change of AFLMBs and LMBs during electrochemical cycling using in situ pressure sensors. The pressure and thickness changes were 423 N and 86.33 μm in LMBs, which were much higher than those of AFLMBs (291 N and 59.39 μm, Figure 2B). These results imply that the electrodeposited Li on the Cu current collector had a relatively dense and chunky morphology compared to Li electrodeposited on Li metal. Optical microscopy (OM) and scanning electron microscopy (SEM) also confirmed that Li deposition on Cu current collectors was more favorable and non‐dendritic than Li deposition on Li anodes (Figure 2C). These results occur because the electrochemical Li deposition is strongly influenced by the dominant facet of the mother substrate. The dominant facet of commercial Li foil is (200), but Li plating on the (110) facet provides much higher electrochemical reversibility than Li plating on (200) of a Li substrate.^[^ ^85^ ^]^ On the other hand, electrodeposited Li on the Cu substrate preferentially grows with (110) facet with large 3D particle sizes, which is advantageous to higher E_v_ and electrochemical reversibility. However, AFLMBs exhibit severely inferior cyclability compared to LMBs owing to the lack of excess Li in the anode; therefore, they adopted an over‐lithiated cathode (Li_2_Ni_0.8_Co_0.1_Mn_0.1_O_2_) to replenish the irreversible Li loss. The Cu||Li‐rich NCM pouch cell achieved 976 Wh L^−1^, which is much higher than that of Li||NCM (846 Wh L^−1^), confirming that AFLMBs are a superior system in terms of the *E_v_* in a practical high‐energy pouch cell. Furthermore, the swelling rate of the anode‐free lithium metal pouch cell was also much slower than that of LMBs. As such, the properties of the current collector greatly affect not only the cyclability of the AFLMBs but also the *E_v_*. As a result, controlling the morphology of electrodeposited Li through current collector modification has attracted significant attention in the research field of AFLMBs.^[^ ^86^, ^87^, ^88^, ^89^ ^]^ We will categorize the currently published articles on current collector engineering into two categories: lithiophilic current collectors and artificial coatings, and we will describe each in detail.

### 2.2 Lithiophilic current collectors

**FIGURE 3:**
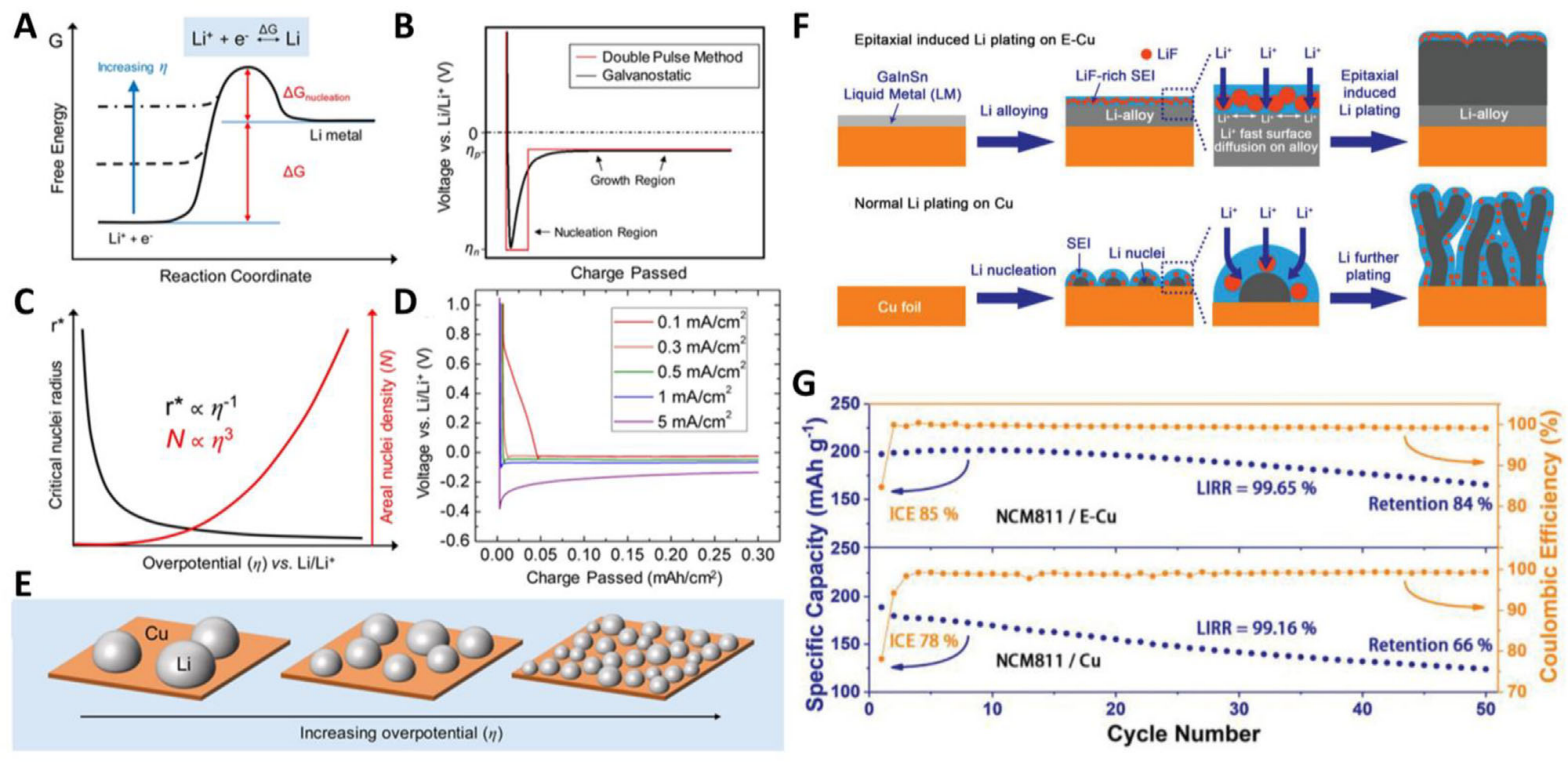
Working principle and representative lithiophilic current collectors for anode‐free lithium metal batteries (AFLMBs). (A) Free energy schematic showing the effects of increasing overpotential on the nucleation energy barrier. (B) Schematic plot comparing the typical voltage profiles of galvanostatic Li deposition (black) and double pulse potentiostatic Li deposition (red). (C) Schematic plot of the dependence of critical Li nuclei radius and areal nuclei density on the overpotential of Li deposition. (D) Experimental voltage profiles of Li deposition on Cu at different current densities for a total capacity of 0.3 mA h cm^−2^. (E) Schematic illustrating the size and density of Li nuclei deposited on Cu at varying overpotentials. Reproduced with permission.^[^ ^89^ ^]^ Copyright 2017, American Chemical Society. (F) Schematic illustration of epitaxial induced Li plating. (G) Cycling performances of 120 mA h level anode‐free lithium metal battery pouch‐cell with E‐Cu. Reproduced with permission.^[^ ^84^ ^]^ Copyright 2021, Wiley‐VCH.

Overpotential is an important factor for non‐dendritic Li growth. Pei et al. revealed the correlation between the overpotential (*η*) and the critical radius of Li nuclei and areal nuclei density through classical nucleation theory and electrochemical experiments (Figure 3A).^[^ ^89^ ^]^ The nucleation barrier for electrodeposition can be effectively changed by adjusting the electrochemical supersaturation at the working electrode and the overpotential of the reduction reaction. During electrodeposition, two major overpotentials were observed: nucleation overpotential (*η_n_*) and plateau overpotential (*η_p_*, Figure 3B). The nucleation overpotential indicates that the potential of the working electrode (Cu) drops sufficiently to drive the nucleation of Li embryos, and the plateau overpotential represents the continuous growth of Li embryos. The formation of a stable embryo of Li atoms is less favorable and has a higher energy barrier than the addition of a Li adatom to existing Li nuclei; thus,—η_p_ is always smaller than—η_n_. According to the classical nucleation theory, the critical radius of nuclei (*r_crit_*) is inversely proportional to nucleation overpotential (*r_crit_* = 2*ϒV_m_*/*F*|*η_n_* |, where *ϒ* = surface energy of the Li‐electrolyte interface, *F* = Faraday constant, and *V_m_* = molar volume of Li).

The nucleation and plateau overpotentials could be adjusted by controlling the current density in the Li||Cu half‐cell configuration because both overpotentials increased significantly as the current density increased (Figure 3C). The authors demonstrated from SEM images after electrodeposition of Li nuclei under different current densities that the radius of the electrodeposited Li nuclei is inversely proportional to an increase in current density, that is, an increase in the plateau overpotential (Figure 3D). In order to achieve non‐dendritic Li growth, larger and fewer Li nucleations are more favorable than smaller and denser Li nucleations, as shown in the left illustration in Figure 3E. In the same context, current collector engineering for reducing the overpotential (both *η_n_* and *η_p_*) is considered to be one of the key strategies for inducing Li plating with a large‐grain and chunky morphology.

A representative strategy to lower the nucleation overpotential and induce lateral Li growth is to introduce elements capable of forming alloys with Li, such as Au,^[^ ^90^, ^91^ ^]^ Ag,^[^ ^92^, ^93^ ^]^ Sn,^[^ ^94^, ^95^ ^]^ Zn,^[^ ^96^, ^97^ ^]^ and Mg^[^ ^98^, ^99^ ^]^. Yan et al. investigated the nucleation overpotential of 11 elements (Au, Ag, Zn, Mg, Al, Pt, Si, Sn, C, Cu, and Ni). In the case of Au, Ag, Zn, and Mg, the nucleation overpotential is nearly zero because they can form a solid solution at room temperature, which was confirmed by the phase diagram of Li–Au. In the case of Pt and Al, the nucleation overpotentials were observed to be 8 and 5 mV at 10 μA cm^−2^, respectively, because both elements have relatively low solubility in Li metal. In contrast, Cu and Ni, which cannot form an alloy with Li, show a clear overpotential of approximately 30 mV. C, Sn, and Si can form an alloy with Li metal but cannot be soluble in Li metal, resulting in a nucleation overpotential of ≈15 mV at the same current density. In this context, researchers have attempted to modify Cu current collectors by introducing lithiophilic elements such as Au,^[^ ^100^ ^]^ Ag,^[^ ^101^, ^102^, ^103^, ^104^, ^105^ ^]^ Sn,^[^ ^49^, ^106^, ^107^ ^]^ Zn,^[^ ^108^ ^]^ SiO*_x_*,^[^ ^69^ ^]^ Te,^[^ ^109^ ^]^ and GaInSn^[^ ^110^ ^]^ to enhance the electrochemical performance and Li plating morphology of AFLMBs.

Lin et al. reported an epitaxially‐induced plating current collector (E‐Cu) by coating Cu current collectors with a liquid metal (Ga:In:Sn = 68.5:31.5:10).^[^ ^110^ ^]^ All three metals can form an alloy with Li metal, resulting in a lower nucleation overpotential. Furthermore, the alloying potential during Li deposition is approximately 0.75 V (vs Li^+^/Li), resulting in a large proportion of the LiF‐rich SEI layer. The coating layer on E‐Cu also enabled the rapid surface diffusion and charge transfer of Li^+^, which led to non‐dendritic Li growth (Figure 3F). As a result, the Li growth morphology and electrochemical performance of E‐Cu were highly enhanced compared to those of Cu. In particular, the CEs, including initial CE (ICE) and CE were largely increased in Li||Cu half‐cell tests with practical operating conditions (current density = 0.5 mA cm^−2^, capacity = 5 mA h cm^−2^). To prove the practical application of E‐Cu, the authors fabricated a 120‐mA h scale anode‐free lithium metal pouch cell. Likewise, the ICE and capacity retention after 50 cycles of E‐Cu were recorded as 85% and 84%, respectively, but that of bare Cu only exhibited 78% and 66%, and the nucleation overpotential of practical anode‐free lithium metal full‐cell was highly improved (Figure 3G). Benefiting from the low mass loading (0.14 mg cm^−2^) of the liquid metal and anode‐free configuration, the energy density of multi‐layer E‐Cu||NCM811 reached 420 Wh kg^−1^. Zheng et al. recently introduced a chemically lithiated Li_4.4_Sn lithiophilic layer for practical AFLMBs. Initially, tin‐plated copper foils were produced by conventional electroless plating methods,^[^ ^49^ ^]^ then Sn@Cu current collectors were dipped into a Li‐biphenyl solution, which caused chemical lithiation to form a Li–Sn alloy.^[^ ^111^ ^]^ Because Sn has high lithiophilicity and alloyability with Li, both the nucleation and plateau overpotentials were improved. In addition, Li_4.4_Sn@Cu delivered a much higher exchange current density, which implied faster charge transfer at the interface between the electrolyte and electrode than that of bare Cu. The lithiophilic and fast charge transfer kinetics of the Li_4.4_Sn layer induced non‐dendritic and compact Li nucleation and growth, which was confirmed by atomic force microscopy (AFM) and SEM images. Finally, the authors applied Li_4.4_Sn@Cu electrodes in a practical pouch cell, which delivered a capacity of 360 mA h and an energy density of 355 Wh kg^−1^. During the 50th cycle, Li_4.4_Sn@Cu||NCM811 maintained 85.5% of its initial capacity, but Cu||NCM811 only recorded 56.3% because of the uniform and dense deposition of Li, which decreased the side reactions with the electrolyte and thus increased the number of Li‐ions that could return to the cathode. Moreover, the rate capability of the Li_4.4_Sn@Cu||NCM811 full cell was greater than that of the Cu||NCM811 cell because of the rapid interface charge transfer between the electrode and electrolyte. Researchers from Samsung have achieved state‐of‐the‐art all‐solid‐state lithium metal batteries (ASSB) in an anode‐free configuration with an argyrodite‐type sulfide solid electrolyte.^[^ ^105^ ^]^ The authors introduced a thin Ag‐C nanocomposite layer (5–10 μm) as the anode instead of the Li metal. Generally, pristine ASSB pouch cells suffer from nonhomogeneous Li growth and short circuits. In addition, the current collector‐SSE interface cannot maintain sufficient contact, resulting in non‐uniform Li deposition. In contrast, the Ag–C nanocomposite layer lowered the nucleation barrier by generating a Li–Ag alloy. Moreover, the majority of the Ag NPs were found at the bottom, close to the SUS current collector, and the particle size was significantly reduced from the initial size. This suggests that the Ag in the Ag–C nanocomposite layer moved in the direction of the current collector continuously during each cycle and did not return to its initial position. In addition, the Li_9_Ag_4_ phase was observed by XRD after 0.1C charging, and it disappeared and converted to peaks of Ag in the subsequent discharging, which indicated the recrystallization of Ag NPs. Owing to the outstanding lithiophilicity of the Ag–C layer and improvement in Li growth morphology, the 0.6 Ah class prototype pouch cell delivered an *E_v_* of over 900 Wh L^−1^ and superior cyclability of over 1000 times.

### 2.3 Artificial coating on the current collector

Because of the highly reactive nature of Li metal and the low lowest unoccupied molecular orbital (LUMO) level of the electrolyte, the electrolytes were chemically and electrochemically degraded to generate a non‐uniform SEI layer during the charging of the AFLMBs. The in‐situ generated SEI layer has inhomogeneity in both chemical species and physical properties; hence, it suffers from uncontrollable Li dendritic growth and rapid cell failure.^[^ ^35^ ^]^ On the other hand, the ex‐situ generated SEI layer, the so‐called artificial SEI layer, can sufficiently provide the properties that the SEI layer should have, such as mechanical strength, dielectric constant, ionic conductivity, spatial homogeneity, fast ionic conductivity, and low interfacial resistance. Various materials have been adopted for artificial coating for many different purposes; therefore, we will discuss representative substances among them.

#### 2.3.1 Polymers

**FIGURE 4:**
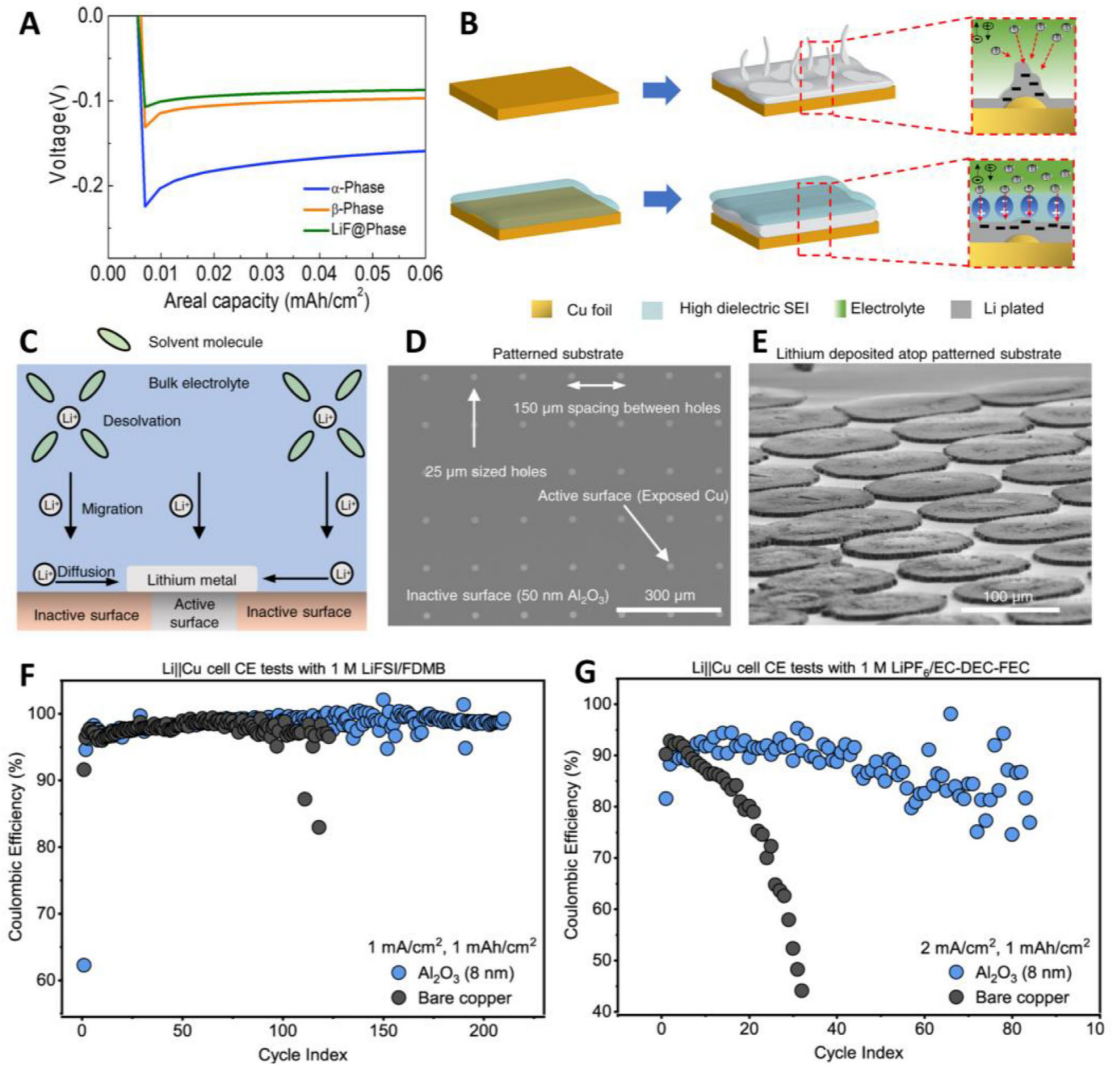
Representative strategies of artificial coatings on current collectors for anode‐free lithium metal batteries (AFLMBs). (A) Voltage profiles of Li deposition, showing the interface overpotentials of bare Cu, α‐PVDF, β‐PVDF, and LiF@PVDF. (B) Schematic illustration of Li growth mechanisms under Li‐ion depletion and mitigating the surface concentration difference during concentration polarization. Reproduced with permission. Copyright 2021, American Chemical Society.^[^ ^112^ ^]^ (C) Schematic illustration of Li deposition on an electrical resistive substrate. (D) Optical image of patterned current collectors with 50 nm Al_2_O_3_ on Cu substrate and 25 μm‐sized holes that expose the underlying Cu substrate. (E) SEM image of Li deposits on patterned current collectors with 0.5 mA h cm^−2^ and 1 mA h cm^−2^. (F,G) Normalized discharge capacity and CEs of anode‐free Cu||NMC 532 pouch cells. Reproduced with permission. Copyright 2022, Springer Nature,^[^ ^115^ ^]^

Assegie et al. introduced a polyethylene oxide (PEO) film onto Cu current collectors for AFLMBs.^[^ ^51^ ^]^ PEO has been widely adopted in the research field of polymer electrolytes because of (1) electrochemical stability, (2) chemical stability with Li metal, (3) flexibility, (4) regulating Li‐ion diffusion, (5) electrically insulating nature, (6) wide potential window, and (7) high dielectric constant for solvation Li‐ions. The authors were motivated by the above merits of PEO, so they fabricated a PEO film by spin‐coating approximately 200 nm with a uniform and defect‐less morphology. The thickness of the PEO film was controlled by the time required for spin‐coating. A thicker and non‐uniform PEO coating layer delivered poor electrochemical performance in the Li||Cu half‐cells compared to a thickness of 200 nm, resulting from the inhomogeneous morphology and defects of the thick PEO film. Active copper may be exposed at the PEO film defect sites, causing severe Li deposition and dendrite growth. As a result, the anode‐free lithium metal full cells with bare Cu and PEO@Cu (200 nm) were tested with LFP cathode, and ether‐based electrolyte. Consequently, PEO@Cu delivered 15% higher capacity retention at the 100th cycle. polyvinylidene difluoride (PVDF) has also been widely studied because of its high dielectric constant and compatibility with Li metal. Tamwattana et al. introduced not only PVDF, but also LiF nanoparticles to obtain a higher dielectric constant.^[^ ^112^ ^]^ There are three phases of PVDF: *α*, *β*, and *γ*, of which β‐PVDF has the highest polarity and dielectric constant owing to its structural configuration. Furthermore, the addition of LiF nanoparticles induced dielectric interactions between PVDF during the film‐forming process on the Cu current collectors, resulting in improved β‐PVDF yield. The 2.5 μm of LiF@PVDF layer lowered both the nucleation and plateau overpotential during Li deposition, indicating a lower nucleation barrier and faster charge transfer than the α‐ and γ ‐phases of PVDF (Figure 4A). The high‐dielectric layer homogenizes the electric field at the interface and mitigates the local hot spot, resulting in a low overpotential at the interface, which induces nondendritic growth. As shown in Figure 4B, the high dielectric layer homogenizes the electric field at the interface and reduces local hot spots (high local current density), resulting in a low overpotential at the interface and non‐dendritic Li growth. Furthermore, LiF@PVDF||LFP showed better electrochemical performance at 0.5 C and 1 C with an ether‐based electrolyte in an anode‐free lithium metal full cell.

#### 2.3.2 Carbon materials

Carbonaceous materials have also been reported to improve the electrochemical performance of AFLMBs, benefiting from their mechanical strength and flexibility, and Li‐ion diffusion ability. Assegie et al. introduced multilayer‐graphene (MLG) onto electropolished Cu current collectors via chemical vapor deposition (CVD).^[^ ^52^ ^]^ The number of layers was carefully controlled to 1–5 layers of graphene by varying the heating temperature, gas flow rate (CH_4_/H_2_), and growth time. Both the nucleation and plateau overpotentials were enhanced owing to the interfacial stability and ability to distribute the local current density of the MLG. In addition, the cyclability of the MLG‐protected anode was improved compared to bare Cu and single‐layer graphene in Li||Cu half‐cells and Cu||LFP full‐cells. In particular, the discharge capacity of MLG anodes at the 100th cycle was approximately 92.62 m Ah g^−1^, which corresponds to 61.34% of initial discharge capacity and ≈99% of CE, whereas, bare Cu only delivered 46% of initial capacity after 100 cycles. The electrodeposited Li metal on the MLG anodes showed a nondendritic, chunky morphology, while bare Cu had highly dendritic, porous features. Furthermore, spin coating,^[^ ^50^ ^]^ capillary liquid bridge,^[^ ^113^ ^]^ and micro‐patterning^[^ ^114^ ^]^ were also adopted to introduce a graphene‐like carbon layer for controlling the Li growth morphology to achieve high‐performance AFLMBs.

#### 2.3.3 Metal oxides

Meanwhile, a metal oxide nanofilm with high electrical resistance has been proposed to control the Li metal deposition morphology (Figure 4C). Oyakhire et al. introduced sub‐10 nm thickness of SnO_2_, ZnO, and Al_2_O_3_ on Cu current collectors via atomic layer deposition (ALD).^[^ ^115^ ^]^ Among them, Al_2_O_3_, which has the highest electrical resistance (22650 Ω/square), operated for more than 300 cycles at 1 mA cm^−2^ and 1 mA h cm^−2^ in the Li||Cu half‐cell, while bare Cu, SnO_2_, and ZnO‐coated Cu only operated under 100 cycles. Unlike the aforementioned discussions about Li nucleation and growth mechanisms, the Li growth morphology and electrochemical performance are greatly improved despite having a very high nucleation overpotential of approximately 1 V owing to the high electrical resistance of the Al_2_O_3_ layer. The authors suggested that the high electrical resistance of Al_2_O_3_ decreases the number of sites for the nucleation of Li metal, inducing sparse nucleation of Li deposits, and that radial diffusion of Li ions towards the nucleated deposits favors lateral growth of Li, inducing dense and low‐surface‐area Li deposits. To demonstrate this hypothesis, they fabricated a patterned substrate with both an inactive surface (50‐nm thick Al_2_O_3_) and an active surface (25 μm sized Cu hole, Figure 4D). It was observed that Li morphologies arose from the active surfaces and spread radially outward into flat, planar, pancake‐like deposits when Li was deposited on top of the patterned substrate in Figure 4E. The authors also extended their results to practical anode‐free lithium metal pouch cells, including a state‐of‐the‐art 1 m LiFSI/FDMB electrolyte and 8‐nm thick Al_2_O_3_ coated Cu current collector. Whereas the cell with bare Cu retained only 40% of its initial discharge capacity of 19.93 mA h after 100 cycles, the Al_2_O_3_‐modified current collectors retained 60% of their initial discharge capacity of 14.72 mA h (Figure 4F,G).

## 3 ELECTROLYTE ENGINEERING

### 3.1 The significance of electrolyte engineering

Modifying current collectors using an artificial layer is a powerful strategy for the uniform deposition and reversibility of Li.^[^ ^48^, ^116^ ^]^ Although a suitable cycle life cannot be retained by current collector modification alone, significant cycle improvement can be obtained when synergized with electrolyte engineering.^[^ ^69^, ^110^ ^]^ During battery operation, electrolytes not only act as charge carriers but also decompose to form an SEI layer, which prevents the decomposition of additional electrolytes and protects Li.^[^ ^117^, ^118^ ^]^ Because no excess Li metal exists in AFLMBs, an SEI layer is firstly formed during the initial charging process. In general, SEI qualities considerably affect the reversibility of Li.^[^ ^76^ ^]^ Formation of a heterogeneous SEI layer causes non‐uniform conduction of Li‐ions, resulting in dendritic and porous Li.^[^ ^43^, ^47^, ^119^, ^120^ ^]^ Consequently, rapid capacity decay and severe safety issues arise. In addition, a large volume change during the stripping/plating process leads to the rupture of the SEI layer, which causes the consumption of fresh Li and electrolyte depletion. Therefore, it is important to form a robust, thin, uniform SEI layer. As the composition and uniformity of the SEI layer significantly depend on the electrolyte, the reversibility of the Li metal is greatly affected by the composition of the electrolyte.^[^ ^76^, ^121^ ^]^

Considering the thermal and chemical stability, and solubility of Li salts, lithium hexafluorophosphate (LiPF_6_), lithium tetrafluoroborate (LiBF_4_), lithium difluoro(oxalato)borate (LiDFOB), lithium bis(trifluoromethanesulfonyl)imide (LiTFSI) and lithium bis(fluorosulfonyl)imide (LiFSI) are widely used in LMBs and AFLMBs.^[^ ^122^ ^]^ These salts lead to inorganic species in the SEI layer such as Li_2_S, LiF, Li_2_O and partially reduced species. These products are dependent on the salts and operating conditions, in which different physicochemical properties are obtained.^[^ ^57^ ^]^ Therefore, mixtures of salts are sometimes used to obtain synergistic effects from different salts.^[^ ^44^, ^122^ ^]^ The decomposition of solvents also constitutes the SEI layer as well, which results in organic species. Though the structure of SEI is not fully understood yet,^[^ ^58^, ^76^ ^]^ it is well‐known that anion‐derived SEI components (i.e. inorganic‐based SEI layer) are in favor of reversible Li stripping/plating compared to those solvent‐derived ones (i.e. organic‐based SEI layer) because of the high (electro)chemical stability and insulating nature of inorganic SEI components, especially LiF.^[^ ^56^, ^121^ ^]^

In this regard, Li‐ion solvation structure engineering is getting tremendous attention.^[^ ^56^, ^123^, ^124^ ^]^ When Li‐ions in the electrolyte electrodeposit on the anode during the charging, desolvation should occur, which involves breaking the interactions between the solute and solvent molecules and removing the solvent molecules from the solvation sheath. Solvents with strong Li‐ion solvation strength, such as 1,2‐dimethoxyethane (i.e. DME) or ethylene carbonate (i.e. EC), form solvent‐rich solvation shells due to strong interactions with Li ions. The free solvent on the electrode surface that occurs during desolvation reacts with the Li metal to form an organic‐rich SEI layer, resulting in poor electrochemical stability. On the other hand, solvents with weak Li‐ion solvation strength increase the interactions between Li ions and anions in the solvation sheath rather than the interactions with the solvent, resulting in anion‐derived, inorganic‐rich SEI layer. In addition to employing solvents with weaker solvation strength, concepts such as high‐concentration electrolyte (HCE) and localized high‐concentration electrolyte (LHCE) are also approaches to increase the amount of anion over solvent in the solvation shell.^[^ ^124^ ^]^

High electrochemical stability and long cycling life can be achieved due to the weak solvation strength, but the resulting ion clustering and low Li‐ion conductivity can lead to poor rate performance,^[^ ^125^ ^]^ so this trade‐off should be addressed through further advanced electrolyte engineering.

### 3.2 HCEs and LHCEs

**FIGURE 5:**
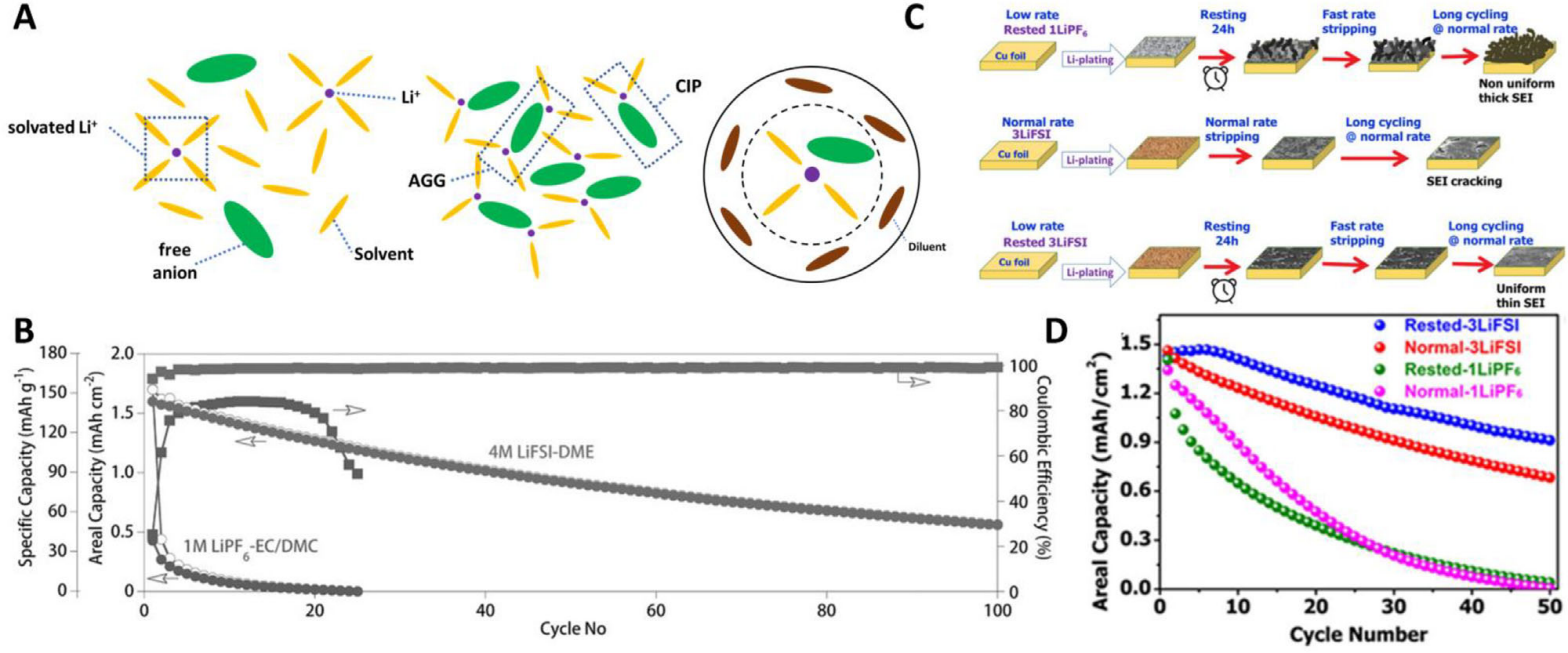
Schematic representation of high‐concentration electrolytes (HCEs) and localized high‐concentration electrolytes (LHCEs), and their applications. (A) Schematic illustration of solvent‐separated ion pairs, contact ion pairs, aggregates and localized high concentration electrolyte. (B) Cycle performance of Cu||LFP anode‐free cells with HCEs and conventional carbonate electrolytes. Reproduced with permission.^[^ ^45^ ^]^ Copyright 2019, Wiley‐VCH. (C,D) Strategy for the formation of robust SEI layer by HCEs with resting protocols and comparison of cycle performance of different SEI formation conditions. Reproduced with permission.^[^ ^74^ ^]^ Copyright 2019, American Chemical Society.

Although the conventional electrolytes 1 m LiPF_6_ dissolved in a mixture of carbonates such as EC, diethyl carbonate (DEC), ethyl methyl carbonate (EMC), or dimethyl carbonate (DMC) have been widely used for LIBs and LMBs because of their high conductivity, oxidative stability, and cost effectiveness,^[^ ^126^ ^]^ high reactivity to Li metal and the formation of a poor SEI layer cause rapid degradation of Li metal. Therefore, ether electrolytes, which are more compatible with Li metal anodes, have been applied to LMBs and AFLMBs.^[^ ^127^, ^128^ ^]^ However, typical ethers, 1,2‐dimethoxyethane (DME) and 1,3‐dixoloane (DOL) are not suitable for high‐voltage cathodes such as NMC, lithium manganese nickel oxide (LMNO), and lithium nickel oxide (LNO) because of oxidative decomposition at ≈4 V.^[^ ^47^, ^125^ ^]^ Therefore, various strategies have focused on simultaneously extending the anodic stability of ethers and building a robust high‐quality SEI layer.^[^ ^47^ ^]^ In Figure 5A, there are many free solvents that do not solvate Li ions at normal electrolyte concentrations (≈1 m). These free solvents are susceptible to oxidation at the cathode and to reduction at the anode. However, as the salt concentration increased (>3 m), most of the solvents are coordinated to Li‐ions, reducing the free solvents. Therefore, HCEs with ether solvents can endure oxidative environments (above 4 V).^[^ ^45^, ^120^ ^]^ In addition, the ratio of anions participating Li‐ion solvation sheath increases, forming contact ion pairs and aggregates for HCEs (Figure 5A). Because anions coordinated with Li ions are more likely to decompose when Li‐ions are reduced, the anion‐derived SEI greatly improves the Li plating/stripping efficiency.^[^ ^43^, ^129^ ^]^ Qian et al. proved the feasibility of AFLMBs using ether‐based high‐concentration electrolyte, 4 m LiFSI/DME^[^ ^45^ ^]^ motivated by the previous study of their group^[^ ^110^ ^]^ showing that the reversibility of Li||Cu half‐cells was greatly improved by 4 m LiFSI/DME. As shown in Figure 5B, the capacity of Cu||LFP decreased significantly within a few cycles when conventional carbonate electrolytes (1 m LiPF_6_ EC/DMC) were used. Interestingly, 4 m LiFSI/DME improved the CEs (>99%) and achieved high capacity retention of 60% after 50 cycles. This is attributed to the nodular and more compact morphology of plated Li metal compared to carbonate electrolytes owing to the anion‐derived SEI layer. Beyene et al. reported the synergistic effect of 3 m LiFSI DOL/DME (1:1, v/v) with a resting protocol.^[^ ^74^ ^]^ By plating Li at a low current rate and resting it for 24 h, they found that a uniform LiF‐rich robust SEI layer was formed (Figure 5C) during the rest period. However, in the case of 1 m LiPF_6_ EC/DEC (1:1, v/v), a thick organic SEI layer was generated during the rest period because of the decomposition of the free solvents. As shown in Figure 5D, the stability of the cells with the resting protocol was increased owing to the synergistic effect with HCEs when using 3 m LiFSI DOL/DME. In contrast, the cells with 1 m LiPF_6_ EC/DEC exhibited a faster capacity decay with the resting protocol. In addition, Cu||LFP cells with 3 m LiFSI DOL/DME achieved 35% capacity retention after 95 cycles under the condition of 1.0 mA cm^−2^.

**FIGURE 6:**
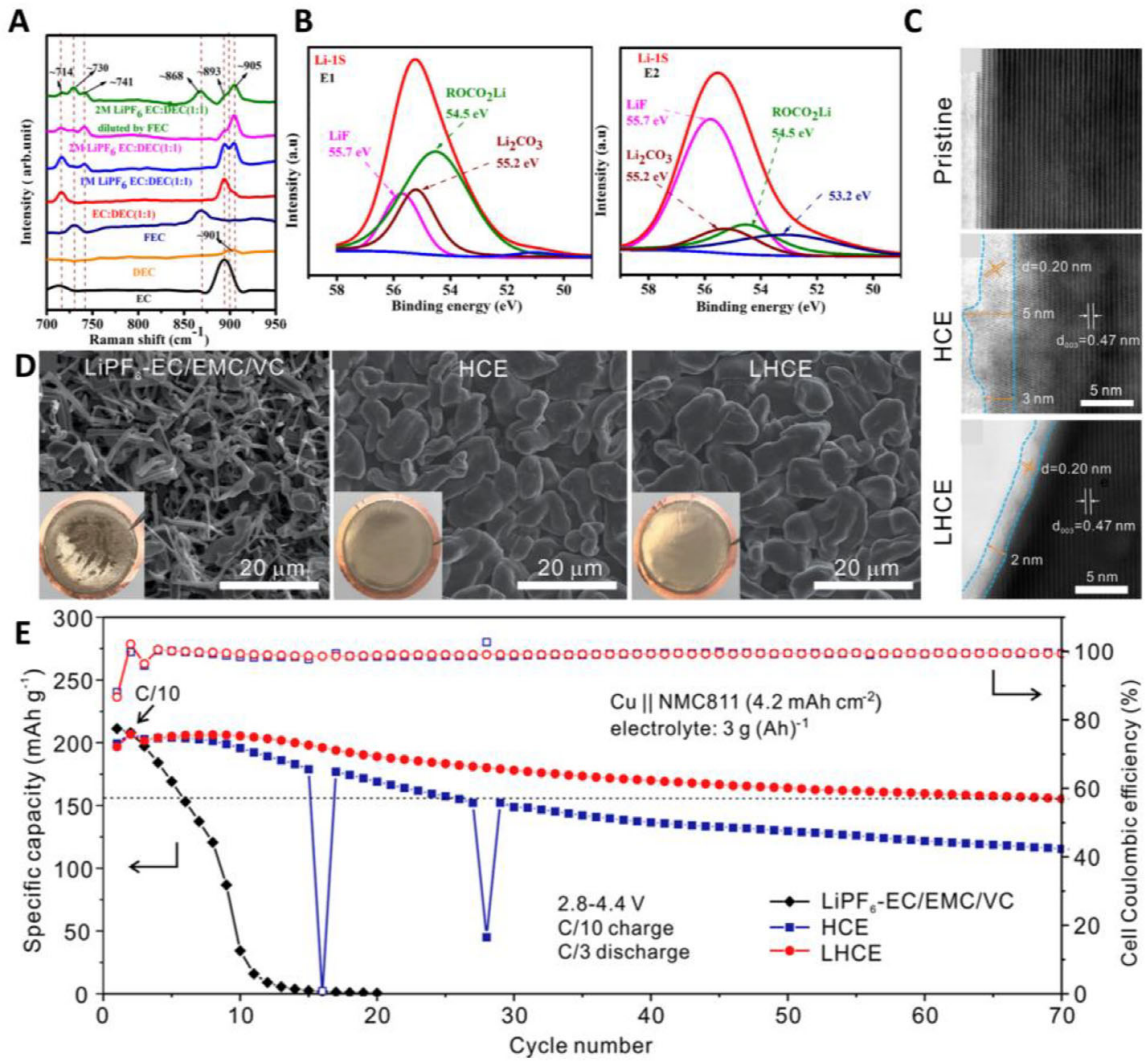
The impact of localized high‐concentration electrolytes (LHCEs) on anode‐free lithium metal batteries (AFLMBs). (A,B) Raman spectra of carbonate electrolytes with different concentrations of LiPF_6_ and diluent, and Li 1s spectra of the surface of current collectors with E1 and E2 electrolytes. Reproduced with permission.^[^ ^119^ ^]^ Copyright 2019, American Chemical Society. (C–E) Annular bright filed scanning electron microscopy image of NCM811 cathodes before and after cycling with HCEs and LHCEs, SEM images of deposited Li metal with conventional carbonate electrolytes (1 m LiPF_6_ in EC‐EMC (3:7 by wt), HCE (LiFSI‐1.2DME), and LHCE (LiFSI‐1.2DME‐3TTE) and cycling performance of Cu||NCM811 anode‐free full cells with lean electrolyte and high loading cathode, 4.2 mA h cm^−2^. Reproduced with permission.^[^ ^43^ ^]^ Copyright 2019, Elsevier.

However, the high viscosity of the HCEs lowers their ionic conductivity and causes less electrode wetting.^[^ ^127^, ^130^ ^]^ In addition, the use of many salts increases the cost.^[^ ^127^ ^]^ To solve these problems, the diluents are mixed with HCEs. Diluents are miscible with the solvent, lowering the viscosity but unable to dissociate the salts, therefore not affecting the Li‐ion coordination structure (Figure 5A). These electrolytes with unique solvation structures are called localized high‐concentration electrolytes, LHCEs. In addition, the wettability and non‐flammability were improved by the addition of diluents.^[^ ^130^ ^]^ These electrolytes with unique solvation structures are called localized high‐concentration electrolytes, LHCEs. Despite the low compatibility of carbonates with Li metal, Hagos et al. used LiPF_6_ salts with carbonates considering the cost and anodic stability of solvents.^[^ ^119^ ^]^ The addition of fluoroethylene carbonate (FEC) diluent lowered the viscosity of 2 m LiPF_6_ EC/DEC (1:1, v/v) +50% FEC (E2) from 8.571 mPa s to 5.680 mPa s. Using Raman spectroscopy, they found that the Li‐ion solvation structure was not affected by the addition of FEC (Figure 6A). Molecular dynamic (MD) simulations confirmed that the ratio of EC and PF_6_^−^ in the Li‐ion solvation sheath was increased by the FEC diluent. Thus, LiF increased in the SEI layer by the reduction of PF_6_^−^ anions (Figure 6B). As a result, Cu||LiNi_1/3_Mn_1/3_Co_1/3_O_2_ cells with E2 electrolytes attained 40% capacity retention at the 50th cycle, showing a significant improvement in carbonate electrolytes. However, carbonate‐based LHCE electrolytes still exhibit limited cyclability compared to ether electrolytes.^[^ ^43^ ^]^ Ren et al. used 1,1,2,2‐tetrafluoroethyl‐2,2,3,3‐tetrafluoropropyl (TTE) as a diluent in LiFSI/DME (LiFSI‐1.2DME‐3TTE in molar ratio).

Though the addition of TTE reduces the lithium‐ion conductivity by half, the wettability and viscosity are dramatically improved. In addition, ^7^Li nuclear magnetic resonance (NMR) analysis showed that the addition of the diluent did not affect the Li‐ion solvation structure. Furthermore, highly fluorinated TTE not only contributes to the thin inorganic cathode electrolyte interphase (CEI) layer formation at the cathode (Figure 6C), but also promotes further decomposition of anions to form a uniform inorganic SEI at the anode through migration of the LUMO towards the FSI^−^ anions. As shown in Figure 6D, the LiF‐rich SEI layer promoted compact and low‐surface Li metal deposition. Owing to the synergistic effect of TTE on both anode and cathode, Cu||NMC811 cells with lean electrolytes (3 g Ah^−1^) showed 77% capacity retention after 70 cycles and attained a high energy density of 412 Wh kg^−1^ (Figure 6E). To further decrease the solvent‐derived SEI layer and promote anion decomposition, Huang et al. rationally designed the LHCEs, which are composed of LiFSI, TTE, and 2‐methyl‐tetrahydrofuran (MeTHF) rather than DME.^[^ ^124^ ^]^ Because of relatively high LUMO level energy, the low polarity of MeTHF, and the enhanced cation‐anion pair, a dense inorganic‐rich inner SEI layer is promoted. As a result, they found that a robust SEI layer effectively prevents the formation of dead Li and SEI reconstruction. Anode‐free pouch cells (Cu||LFP) modified with their electrolyte showed 41.6% capacity retention after 150 cycles.

### 3.3 Dual‐salt electrolytes

Compared to a single salt, the co‐existence of anions changes the quality of the SEI layer by the physical properties and decomposition chemistry of each salt, resulting in significant effects on Li metal protection.^[^ ^120^, ^131^ ^]^ Motivated by the synergistic effect of dual salts, Beyene et al. mixed relatively inexpensive LiTFSI with LiFSI and dissolved them in DOL/DME (1:1, v/v) to synthesize dual‐salt electrolytes.^[^ ^132^ ^]^ They determined that LiFSI is more susceptible to reduction than LiTFSI, which is responsible for the development of ionic conductive inorganic species (LiF and Li_2_O) through preferential FSI^−^ decomposition. In addition, LiTFSI improved ionic conductivity. Owing to the synergistic effect of the binary salts LiFSI and LiTFSI, a compact and robust SEI was formed, improving the reversibility of Li. Xu and co‐workers reported a high concentration of LiFSI and LiTFSI dissolved in DME (4.6 m LiFSI + 2.3 m LiTFSI in DME, BSEE) to form a stable electrolyte even at high voltage (4.4 V) while stabilizing the plating/stripping of Li metal.^[^ ^120^ ^]^ It was found that the conductivity was not significantly different from the reference 1.0 m LiPF_6_ EC/EMC (3:7, v/v), Gen 2, even when a high concentration of dual‐salt was used. Furthermore, LiTFSI prevented the precipitation of LiFSI. From the X‐ray photoelectron spectroscopy (XPS) spectra, they found that more Li_2_O and Li_2_S appeared in the single salt electrolyte, 4.6 m LiFSi‐DME (SSEE) due to the decomposition of LiFSI during the initial cycle and after 200 cycles. However, these species appear less in BSEE because of the presence of LiTFSI. Initially, LiTFSI was decomposed, forming a C–F moiety and further reducing to LiF after cycling. Combined with computational simulations, they found that TFSI^−^ anions affect the decomposition kinetics of FSI^−^ anions, and a uniform and robust SEI layer is formed compared to the fast FSI^−^ decomposition. The oxidation stability of BSEE increased up to approximately 5 V by linear sweep voltammetry (LSV) analysis and was even more stable than Gen 2. As a result of the synergistic effect of dual salts, Cu||NMC622 anode‐free full cells operated at C/10 and D/3 with BSEE had a high ICE as well as capacity retention of 90.9 mA h g^−1^ and high CE of 98.6 % after 54 cycles. In particular, in tests with various compositions, while maintaining a constant total salt concentration, it was found that an appropriate ratio of LiFSI to LiTFSI severely affects the reversibility of Li. In 2019, Dahn's group performed impressive work by combining dual salts in carbonates.^[^ ^44^ ^]^ They designed practically concentrated dual salts (LiDFOB and LiBF_4_) dissolved in FEC:DEC (1:2, v/v). Anode‐free pouch cells with 1 m LiDFOB 0.2 m LiBF_4_ delivered 80 % initial capacity for 90 cycles, outperforming the single salt cells and other combinations of salts. This outstanding improvement in the capacity retention of the dual‐salt electrolyte was attributed to the formation of compact bulky Li metal. Unlike fluorine 1s spectra of the cycled anode from cells with LiPF_6_, not only LiF but also the large amount of organic fluorine components which are decomposed byproducts of LiDFOB were found for those with LiDFOB and LiBF_4_.This difference in SEI composition induces a large change in the deposition morphology of Li. However, the continuous decomposition of salts eventually causes cell degradation. In 2020, Dahn's group investigated the electrolyte degradation of dual‐salt electrolytes and developed optimized electrolytes.^[^ ^133^ ^]^ They found that a favorable plated Li morphology can be ascribed to SEI layer formation by the continuous decomposition of salts. According to the suggested mechanism of salt decomposition by authors, the oxidation of LiDFOB in the presence of FEC solvents at the positive electrode builds an Li‐ion conductive CEI layer. Unreacted LiDFOB generates LiBF_4_ by reacting with polymeric byproducts of LiDFOB and FEC, which is beneficial to the Li anode.^[^ ^134^ ^]^ In addition, the reduction of FEC makes CO_2_ and LiF.^[^ ^135^, ^136^ ^]^ In order to compensate for the consumption of salts, they used high concentration dual‐salt electrolytes by combining 2 m LiDFOB and 1.4 m LiBF_4_ in FEC/DEC (1:2, v/v). With hot formation cycles and pressurized conditions,^[^ ^66^, ^137^ ^]^ anode‐free pouch cells with high concentration dual salt electrolytes delivered 80% of initial capacity after 200 cycles.

### 3.4 Solvents modification

**FIGURE 7:**
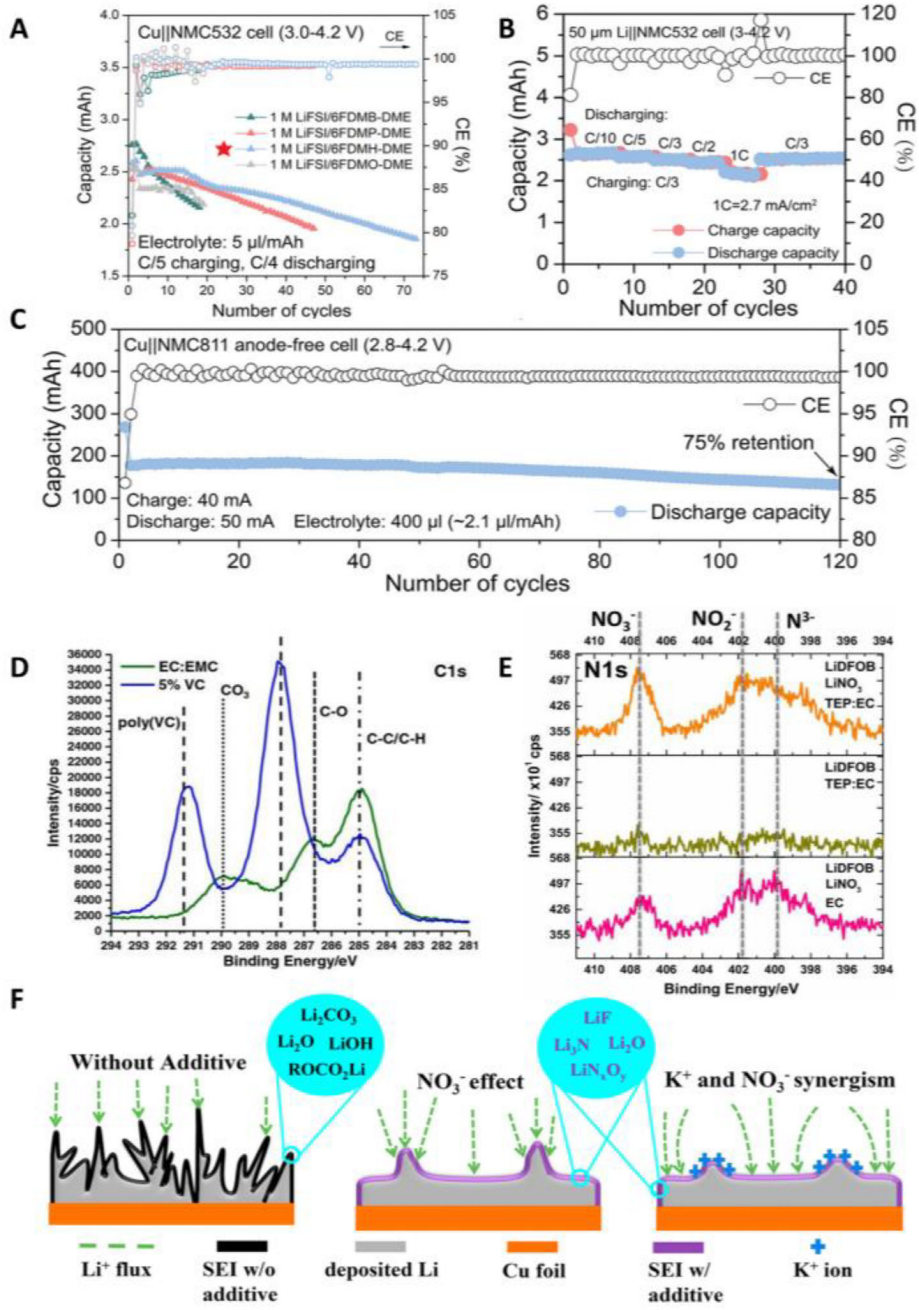
Representative studies on solvent modifications and additives for anode‐free lithium metal batteries (AFLMBs). (A–C) Cu||NMC532 full‐cell cycle performance of various electrolytes with modified solvents by fluorination and chain length, rate performance of 1 m LiFSI/6FDMH‐DME electrolytes and high cycle stability of anode‐free Cu||NMC532 pouch cells achieving 75% of initial capacity retention at 120 cycles. Reproduced with permission.^[^ ^125^ ^]^ Copyright 2021, Wiley‐VCH. (D) Change of C 1s spectra of SEI composition by using VC. Reproduced with permission.^[^ ^143^ ^]^ Copyright 2017, The Electrochemical Society. (E) NO_3_^−^ derived SEI layer in N 1s spectra. Reproduced with permission.^[^ ^145^ ^]^ Copyright 2019, The Electrochemical Society. (F) Dual functional effects of KNO_3_ additive in the stabilization of Li metal. Reproduced with permission.^[^ ^149^ ^]^ Copyright 2019, Elsevier.

HCE and LHCE changed the Li‐ion solvation structure, resulting in an anion‐derived SEI. This Li‐ion‐conductive SEI layer is conducive to compact and uniform Li deposition. However, a large amount of salt usage increases the cost, and the diluent, which takes a large portion of electrolytes, increases the ionic resistance and often requires unstable solvents for Li‐ion solvation.^[^ ^138^, ^139^ ^]^ Therefore, an advanced solvent contributing anion‐rich SEI layer through appropriate Li solvation with a low salt concentration while having little reactivity with Li metal is required. Motivated by the high (electro)chemical stability of alkyl chains and F groups, Yu et al. rationally designed fluorinated solvents.^[^ ^47^ ^]^ First, the alkyl chain of ether was lengthened to synthesize 1,4‐dimethoxylbutane (DMB). They then fluorinated the extended alkyl groups of DMB to produce 1,4‐dimethoxybutane (FDMB). LSV analysis showed that the oxidation stability of LiFSI/FDMB was significantly improved to 6.14 V compared to LiFSI/DME (3.9 V) and LiFSI/DMB (5.2 V). Furthermore, MD simulations revealed that Li‐ion binds to O, and F weakly in FDMB. Owing to the unique solvation structure of FDMB, the ratio of FSI^−^ to solvent in the first solvation shell of Li‐ion was 3.29, which was significantly higher than that of DME (2.31) and DMB (2.29). This leads to a thin (≈6 nm) and homogeneous SEI, which benefits Li‐ion conduction. Owing to high compatibility with Li metal and oxidation stability of FDMB, Cu||NMC532 anode‐free pouch cells delivered 80% capacity retention after 100 cycles (CE, 99.98%). Cui's group further extended the fluorinated chains of FDMB to improve the stability of Li metal and oxidation.^[^ ^125^ ^]^ However, the low Li‐ion solvating ability of solvents with more ─CF_2_─ groups reduces the ionic conductivity. Because of the low Li‐ion solvation power of FDMB analogs, DME was mixed to reduce the ionic resistance. Considering the stability of the electrolytes and ionic conductivity, 1,6‐dimethoxyhexane (FDMH), which has two more ─CF_2_─ chains than FDMB with DME (LiFSI/6FDMH‐DME, *v_FDMH_*:*v_DME_* = 6:1) was chosen (Figure 7A). The optimized LiFSI/6FDMH‐DME showed a high oxidation stability of over 6 V and low interfacial resistance owing to the synergistic effects of FDMH and DME. MD simulations revealed that both FDMH and DME participate in Li‐ion solvation, which is different from the concept of LHCEs in that FDMH participates in Li‐ion solvation. As shown in Figure 7B, Li||NMC532 shows high rate performance with high capacity retention (over 80%) at 1 C and excellent reversibility as well due to improved kinetics of Li deposition. In addition, anode‐free Cu||NMC811 pouch cells achieved 75% capacity retention after 120 cycles (Figure 7C).

### 3.5 Additives

Among various purposes of additives such as passivation of electrodes, physical property improvement, and securing safety,^[^ ^140^, ^141^, ^142^ ^]^ formation of a high‐quality SEI layer is focused on AFLMBs. With small amounts (typically, less than 10% in volume or weight), additives decompose electrochemically and chemically by reacting with Li metal leaving the passivation layer. Vinylene carbonate (VC) is known for improving the stability of the anode by forming a polymeric SEI, which suppresses the volume expansion. Brown et al. investigated the effect of VC in anode‐free Cu||LFP cells by adding 5 wt% VC to 1.2 m LiPF_6_ EC:EMC (3:7, vol.).^[^ ^143^ ^]^ As shown in Figure 7D, the main SEI components changed from lithium ethyl dicarbonate, Li_2_CO_3_, and LiF to poly(VC) when VC additive was added. These polymeric SEI components improve the reversibility of Li metal in carbonate electrolytes. Similarly, LiNO_3_ is a widely used additive in LMBs because of the high ionic conductivity of the N species in the SEI layer (Li_3_N, Li*_x_* NO*_y_*).^[^ ^144^ ^]^ Motivated by the beneficial effects of LiNO_3_, Brown et al. dissolved LiNO_3_ in carbonates.^[^ ^145^ ^]^ To increase the solubility of LiNO_3_ in carbonates, they added triethyl phosphates (TEP) (1 m LiDFOB + 0.2 m LiNO_3_ TEP/EC/DMC (8.4:8.4:83.2, v/v/v)). As shown in the N 1s spectra (Figure 7E), NO_3_^−^, NO_2_^−^ and N^3−^ species were detected on the cycled Li metal by the decomposition of LiNO_3_. As a result, compared to the baseline electrolyte (1 m LiDFOB EC/DMC (16.8:83.2, v/v)), the modified electrolytes with LiNO_3_ and TEP doubled the cycle stability of anode‐free cells by the favorable effects of N species stabilizing the Li metal. The uneven deposition of Li by tip effects accelerates the dendritic growth of Li.^[^ ^146^, ^147^ ^]^ Alkali metal (Cs^+^, K^+^, Na^+^) ions have been exploited as additives for LMBs.^[^ ^60^, ^148^ ^]^ During Li deposition, alkali metal ions are aggregated at the tip where the electric field is concentrated and repel Li ions through electric repulsion preventing tip growth (Figure 7F). Synergistically, anions decompose to form a Li ion‐conducting SEI layer. Sahalie et al. used KNO_3_ as an additive in 1 m LiPF_6_ EC/DEC.^[^ ^149^ ^]^ With the advantages of shielding effect of K‐ions and formation of favorable SEI from NO_3_^−^ anions, Cu||NMC cells achieved 42% capacity retention after 51 cycles. Furthermore, Hagos et al. used 2 wt% KPF_6_ and 2 vol% tris(trimethylsilyl) phosphite (TMSP) as dual additives to 1 m LiPF_6_ EC/DEC.^[^ ^150^ ^]^ Like KNO_3_ additives, K‐ions prevent the tip growth of Li, and PF_6_^−^ contributes to the Li‐ion conduction interphase. In addition, TMSP suppresses SEI degradation by scavenging HF, which is generated by traces of water and PF_6_^−^.^[^ ^151^ ^]^ Owing to the synergistic effect of dual additives, Cu||NMC cells showed 48% capacity retention after 20 cycles. As stated for electrolyte modification, tremendous efforts have been made to configure the inorganic‐rich SEI by modifying the Li‐ion solvation structure.^[^ ^43^, ^47^, ^120^ ^]^ In 2022, Kim et al. dispersed Li_2_O nanoparticles to 1 m LiPF_6_ in EC/DEC with 10 wt% FEC.^[^ ^152^ ^]^ As shown in Figure 5D, radial distribution functions (RDFs) revealed that the ratio of fluorinated species (PF_6_^−^ and FEC) to non‐fluorinated (DEC and EC) increases around Li_2_O. Owing to the formation of an inorganic‐rich SEI layer by the decomposition of F‐containing species in the Li‐ion solvation sheath, Li_2_O suspension electrolytes stabilize the Li metal with improved CE. With the Li_2_O suspension electrolytes, both the initial and average CEs were improved for the Cu||NMC811 cells. More importantly, the Li_2_O suspension was applicable to recently reported advanced electrolytes (LHCEs and fluorinated solvents).^[^ ^43^, ^47^ ^]^ Although the improvement in CE was small compared to that of carbonate electrolytes, the Li_2_O suspension improved the CE of the cells with advanced electrolytes, suggesting the versatility of suspension electrolytes. Dahn's group explored the effects of conventionally used various additives and co‐solvents on their dual‐salt electrolytes,^[^ ^44^ ^]^ 0.6 m LiDFOB 0.6 m LiBF_4_ in FEC:DEC (1:2, v:v). They reported 65 different electrolyte formulations and compared the total energy delivery over 140 cycles.^[^ ^52^ ^]^ Of the 65 electrolyte additives, only tris (2,2,2‐trifluoroethyl) phosphate (TTFEP), p‐toluene sulfonyl isocyanate (PTSI), 1,5‐dicyano pentane (DCP), and LiClO_4_ showed positive effects, and the other additives deteriorated the performance. A variety of electrolytes can be prepared by the combination of additives and base electrolytes, but the appropriate species and quantity of additives greatly affect the performance of the cells. This shows that electrolyte optimization with additives is a complex and time‐consuming process, and the mechanism is not yet fully understood.

## 4 CATHODE ENGINEERING

Though the limiting factor of the cycling performance of AFLMBs is mainly the anode, electrochemical products of active materials or additives in the cathode would greatly improve the cycle stability. In the case of soluble species to electrolytes originating from the cathode, they diffuse to the anode and affect the composition of the passivation layer resulting in better reversibility of Li plating/stripping. Furthermore, the irreversible decomposition of lithiated compounds embedded in the cathode during the first charge process provides an invaluable Li source in the anode, which replenishes inactive Li. In this section, we focus on the AFLMBs enabled by cathode modification, especially Li_2_S cathode, over‐lithiated cathode, and sacrificial additives.

### 4.1 AFLSBs based on Li_2_S cathode

**FIGURE 8:**
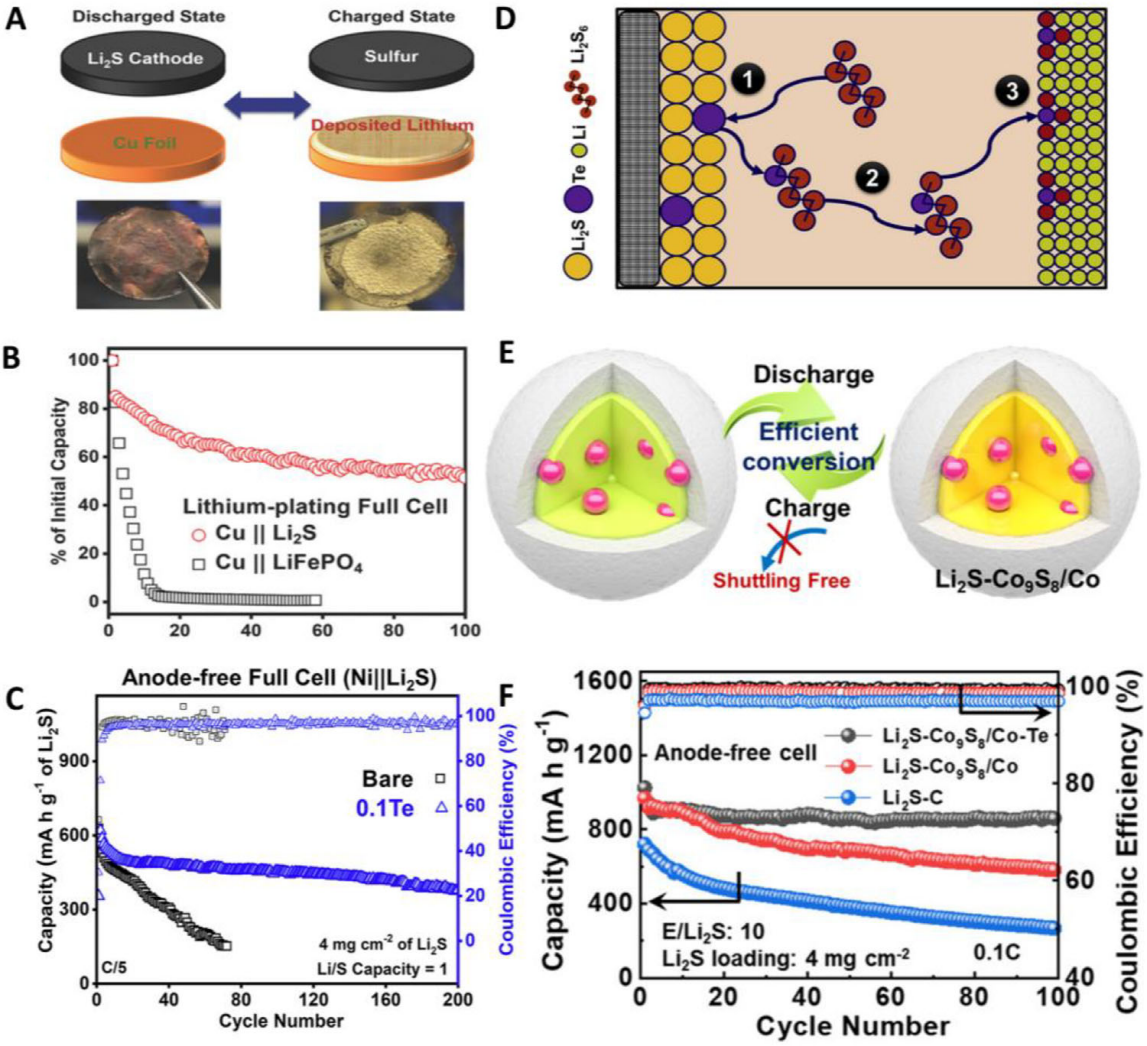
Schematic illustrations of anode‐free lithium‐sulfur batteries (AFLSBs) and their representative studies. (A,B) AFLSB cell configurations composed of Cu foil anode and Li_2_S cathode and higher stability of AFLSBs over Cu||LFP full cells. Reproduced with permission.^[^ ^61^ ^]^ Copyright 2018, Wiley‐VCH. (C,D) Effect of Te additives for stabilization of anode and formation mechanism of Tellurium‐based SEI layer on Li metal. Reproduced with permission.^[^ ^63^ ^]^ Copyright 2020, Elsevier. (E,F) Schematic illustration of Li_2_S and catalyst composite (Li_2_S‐Co_9_S_8_/Co) and its advantage and galvanostatic discharge at 0.1 C of AFLSBs with different cathode composites (Li_2_S‐Co_9_S_8_/Co‐Te, Li_2_S‐Co_9_S_8_/Co, and Li_2_S‐C). Reproduced with permission.^[^ ^161^ ^]^ Copyright 2021, American Chemical Society.

Owing to the high price, toxicity, and limited specific capacity (≈200 mA h g^−1^) of intercalation‐type cathodes,^[^ ^153^ ^]^ conversion‐type cathodes have attracted enormous attention.^[^ ^154^, ^155^ ^]^ Among them, lithium sulfide (Li_2_S), which is a fully discharged state of sulfur (S), is an attractive material with a high theoretical specific capacity (1166 mA h g^−1^) and its applicability as a cathode material for AFLMBs.^[^ ^61^, ^156^ ^]^ In 2018, Nanda et al. reported anode‐free lithium‐sulfur batteries (AFLSBs).^[^ ^61^ ^]^ AFLSBs were composed of a Li_2_S/CNT cathode with a bare Cu foil (Figure 8A). A significantly different capacity retention of the Li_2_S/CNT cathode was observed compared to that of the LFP cathode in Figure 8B. The capacity of Cu||LFP cathode decreased rapidly within a few cycles (2.2% after 10 cycles). On the other hand, Li_2_S/CNT cathode retained 51.5% of its initial capacity even after 100 cycles. This excellent cycle performance of the AFLSBs is attributed to the intrinsic properties of stabilizing the Li metal in the (AF)LSBs.^[^ ^157^ ^]^ During the charge and discharge processes of (AF)LSBs, intermediate polysulfide (Li_2_S*_x_*, 2 < *x* ≤ 8) species diffuse to the anode and reduce to ionic conductive Li_2_S and Li_2_S_2_ by reacting with Li metal and stabilizing Li deposition.^[^ ^158^ ^]^ The use of Li_2_S as an active material for anode‐free systems is not only advantageous in terms of capacity but also greatly stabilizes the stripping and plating of Li metal. Although AFLSBs have shown higher cyclability than AFLMBs with NMC and LFP cathodes, it is still difficult to achieve more than 100 cycles with proper capacity retention without modification. To improve the reversibility of the Li metal of AFLSBs, Manthiram's group incorporated 10 wt% (vs Li_2_S) of Te as a cathode additive.^[^ ^63^ ^]^ Anode‐free Ni||(Li_2_S+0.1 Te) full cells at C/5 attained 50 % capacity retention after 240 cycles (Figure 8C). On the other hand, in the case of AFLSBs without Te, the capacity retention dropped to 50 % after only 34 cycles. The improved cycle stability with the Te additive is attributed to the formation of soluble polytellurosulfides (Li_2_Te*_x_* S*_y_*), which are derived from the reaction between Te and lithium polysulfides. As illustrated in Figure 8D, these soluble Li_2_Te*_x_* S*_y_* diffused to the anode and reacted with Li metal, forming Li_2_TeS_3_ and Li_2_Te on the Li metal anode. The covalence of the Te–S bonds increases the Li‐ion diffusivity through the SEI layer. Te additives are advantageous for improving the stability of Li metal by simply mixing Te with Li_2_S without complex modifications of the anode or cathode owing to the unique characteristics of LSBs. To realize high‐performance AFLSBs, not only the stability of the anode, but also the problems of the cathode, such as the low electrical conductivity of Li_2_S, low kinetic shuttling of lithium polysulfides, and low utilization of Li_2_S must be solved simultaneously.^[^ ^62^, ^159^, ^160^ ^]^ In this regard, He et al. synthesized an Li_2_S/electrocatalysts (Li_2_S‐Co/Co_9_S_8_) nanoparticle composite via the carbothermal reduction reaction of Li_2_SO_4_ and CoSO_4_ (Figure 8E).^[^ ^161^ ^]^ Nano‐sized Co‐based catalysts in intimate contact with Li_2_S accelerate the polysulfide conversion reaction and suppress the shuttle effect, resulting in a high Li_2_S utilization ratio. Finally, they introduced Te^[^ ^63^ ^]^ into the Li_2_S−Co_9_S_8_/Co cathode to stabilize the anode (Li_2_S−Co_9_S_8_/Co−Te). With high Li_2_S loading (4 mg cm^−2^) at 0.1 C, they compared the performance of Li_2_S−Co_9_S_8_/Co−Te to Li_2_S–C (without electrocatalyst and Te) and Li_2_S−Co_9_S_8_/Co (without Te) cathodes (Figure 8F). As expected, Li_2_S−Co_9_S_8_/Co−Te cathode delivered a high initial capacity of 1025 mA h g^−1^ and low capacity decay (84% capacity retention after 100 cycles). Surprisingly, anode‐free lithium‐sulfur pouch cells modified with Li_2_S−Co_9_S_8_/Co−Te under practical conditions (Li_2_S, 4 mg cm^−2^ and lean electrolyte 4.5 μL mg^−1^) achieved promising results with a high energy density of 221 W h kg^−1^. To enhance the cyclability and capacity of AFLSBs, it is necessary not only to stabilize the anode, but also to prevent the dissolution of polysulfide and Li_2_S occurring at the cathode at the same time.

### 4.2 Li reservoir

**FIGURE 9:**
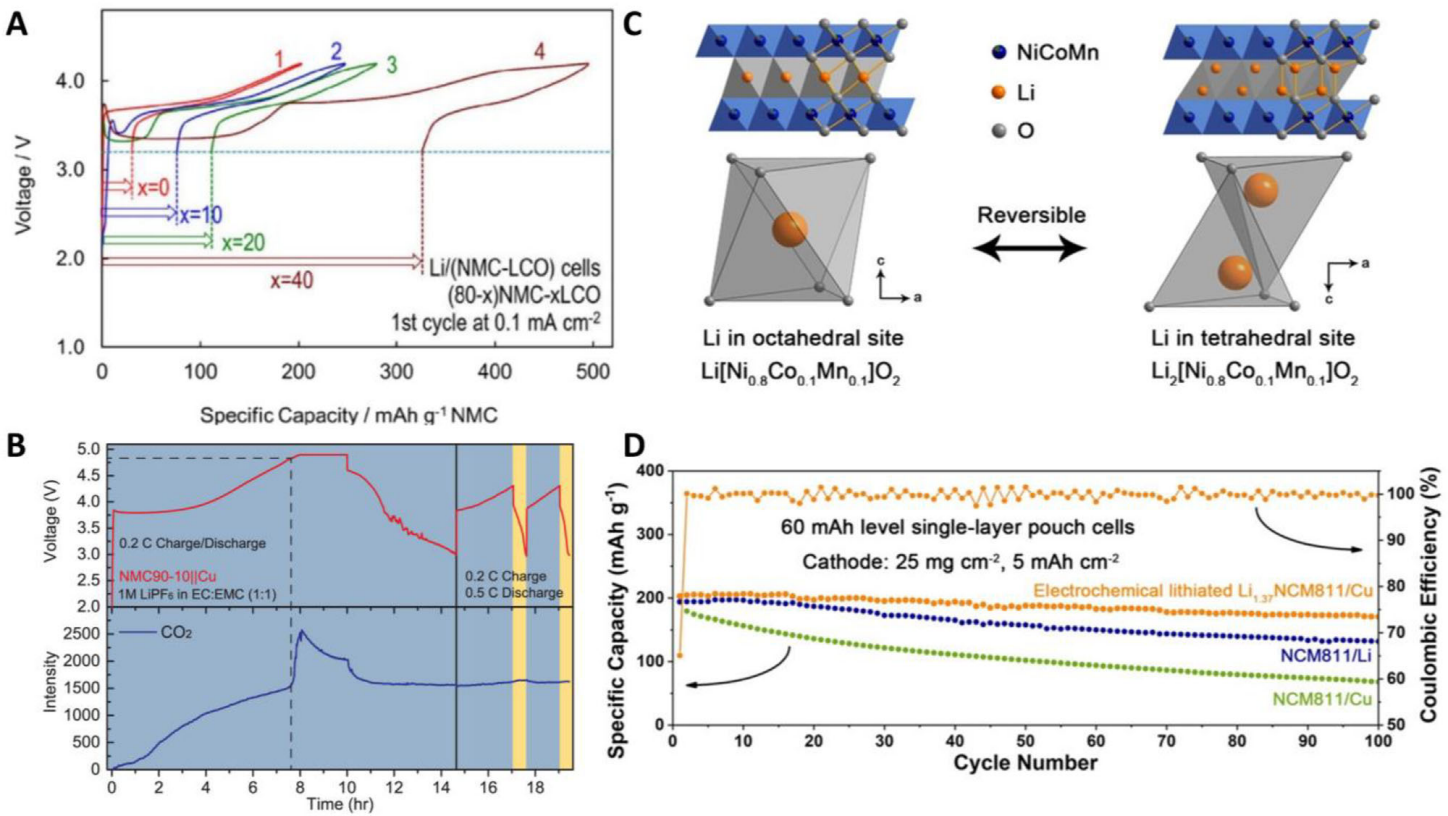
Representative studies on sacrificial cathode additives to provide Li reservoir for anode‐free lithium metal batteries (AFLMBs). (A) Irreversible Li reservoir formation with different ratio of NMC‐LCO at 1st cycle. Reproduced with permission.^[^ ^163^ ^]^ Copyright 2018, Elsevier. (B) Irreversible generation of Li and CO_2_ gas by decomposition of LO at 4.7 V. Reproduced with permission.^[^ ^142^ ^]^ Copyright 2022, American Chemical Society. (C,D) Reversible Li storage from octahedral sites for Li[Ni_0.8_Co_0.1_Mn_0.1_]O_2_ to tetrahedral sites for Li_2_[Ni_0.8_Co_0.1_Mn_0.1_]O_2_ and extended cycle stability of electrochemically lithiated Li_1.37_NCM811/Cu pouch cells over LMBs. Reproduced with permission.^[^ ^71^ ^]^ Copyright 2021, Wiley‐VCH.

Although irreversible Li loss caused by dead Li and SEI formation occurs in the same manner for AFLMBs and LMBs, replenishment of Li loss does not occur in AFLMBs, resulting in a significant difference in cyclability between LMBs and AFLMBs. To compensate for the initial irreversible loss of Li, pre‐lithiation strategies have been widely used in LIBs^[^ ^162^ ^]^ to compensate the Li loss during the initial charge. Similarly, Wang's group demonstrated an in situ‐built Li reservoir during the initial charge.^[^ ^163^ ^]^ They combined a Li‐rich Li_2_CuO_2_ (LCO) additive with NCM811, (80‐*x*)NMC‐*x*LCO. LCO delivered an irreversible capacity of 321 mA h g^−1^ within a potential window of 3.2–4.2 V. As shown in Figure 9A, the Li reservoir was successively built by incorporating LCO; the reservoir size increased as the amount of LCO increased. With two formation cycles at 0.1 mA cm^−2^ for the formation of the Li reservoir, the cycle stability of the Cu/NMC‐LCO cells was largely extended. In 2021, motivated by the sacrificial Li compound strategy, Qiao et al. incorporated lithium oxide (Li_2_O) as a sacrificial agent in the cathode for AFLMBs.^[^ ^64^ ^]^ It should be noted that Li_2_O has been studied extensively in metal‐air batteries and as a sacrificial additive for LIBs because of its high theoretical capacity (O_2_ + 4Li^+^ + 4e^−^ → Li_2_O, 1793 mA h g^−1^).^[^ ^164^, ^165^ ^]^ By mixing 25 wt% Li_2_O with NCM811, an irreversible capacity of 320 mA h g_NCM_^−1^ attributed to the oxidation of Li_2_O appeared during the initial charging process. TTE additives were used to inhibit the oxygen evolution reaction. The nucleophilic reaction of TTE and the superoxide anion (O_2_^−^) prevents oxygen evolution and produces a CEI layer composed of LiF, which further increases the oxidative stability of the ether electrolyte. When fabricated as anode‐free pouch cells with Li_2_O additive, not only a high energy density of 320 W h kg^−1^ was achieved, but also no significant capacity decay was observed over 200 cycles, and 80% of initial capacity was maintained after 300 cycles. The Li donor reacts irreversibly during the first charging process to form a Li reservoir, and it is very effective in increasing the lifespan of AFLMBs by supplementing Li loss. Huang et al. used lithium oxalate (LO) as an additive in the cathode as the sacrificial Li source.^[^ ^65^ ^]^ During initial charging, LO irreversibly oxidizes at 4.7 V to form Li reservoir and carbon dioxide (Figure 9B). As synthesized CO_2_ diffused to the anode and formed a Li_2_CO_3_‐rich SEI layer on the Li metal.^[^ ^166^ ^]^ Furthermore, Dong et al. reported Li_2_CO_3_ additive in AFLMBs. In addition to the formation of a Li reservoir from Li_2_CO_3_ incorporated into the cathode during the initial charge process, Li_2_CO_3_ coated on the anode reacts with PF_5_^−^ resulting in a LiF‐rich SEI layer. The dual function of the additive resulted in anode‐free Cu@Li_2_CO_3_/NCM811@Li_2_CO_3_ cells delivering high capacity retention (81.60%) at 1/3 C after 100 cycles.^[^ ^167^ ^]^ However, the additive takes up weight, which lowers the energy density after the first charging reaction. Therefore, additional additives improve the cycle stability while simultaneously sacrificing the energy density. In the absence of additives, Lin et al. synthesized Li‐rich Li_2_NCM811 (Li_2_[Ni_0.8_Co_0.1_Mn_0.1_]O_2_) to build a Li reservoir in the anode.^[^ ^71^ ^]^ When additional Li ions are introduced into NCM811, they are partially stored in tetrahedral sites and the NMC811 is transformed into P3m1 type Li_2_NCM811 (Figure 9C). After delithiation, the structure reversibly changed to R3m NCM811. With a partially over‐lithiated Li_2_NCM811 cathode, the anode‐free pouch cell composed of Li_1.37_[Ni_0.8_Co_0.1_Mn_0.1_]O_2_ cathode enabled high capacity retention of 84% at 100th cycle and achieved high energy density (447 Wh kg^−1^) in the lean electrolyte (2 g [A h]^−1^) and high loading system (25 mg cm^−2^) in Figure 9D.

## 5 PROTOCOLS

**FIGURE 10:**
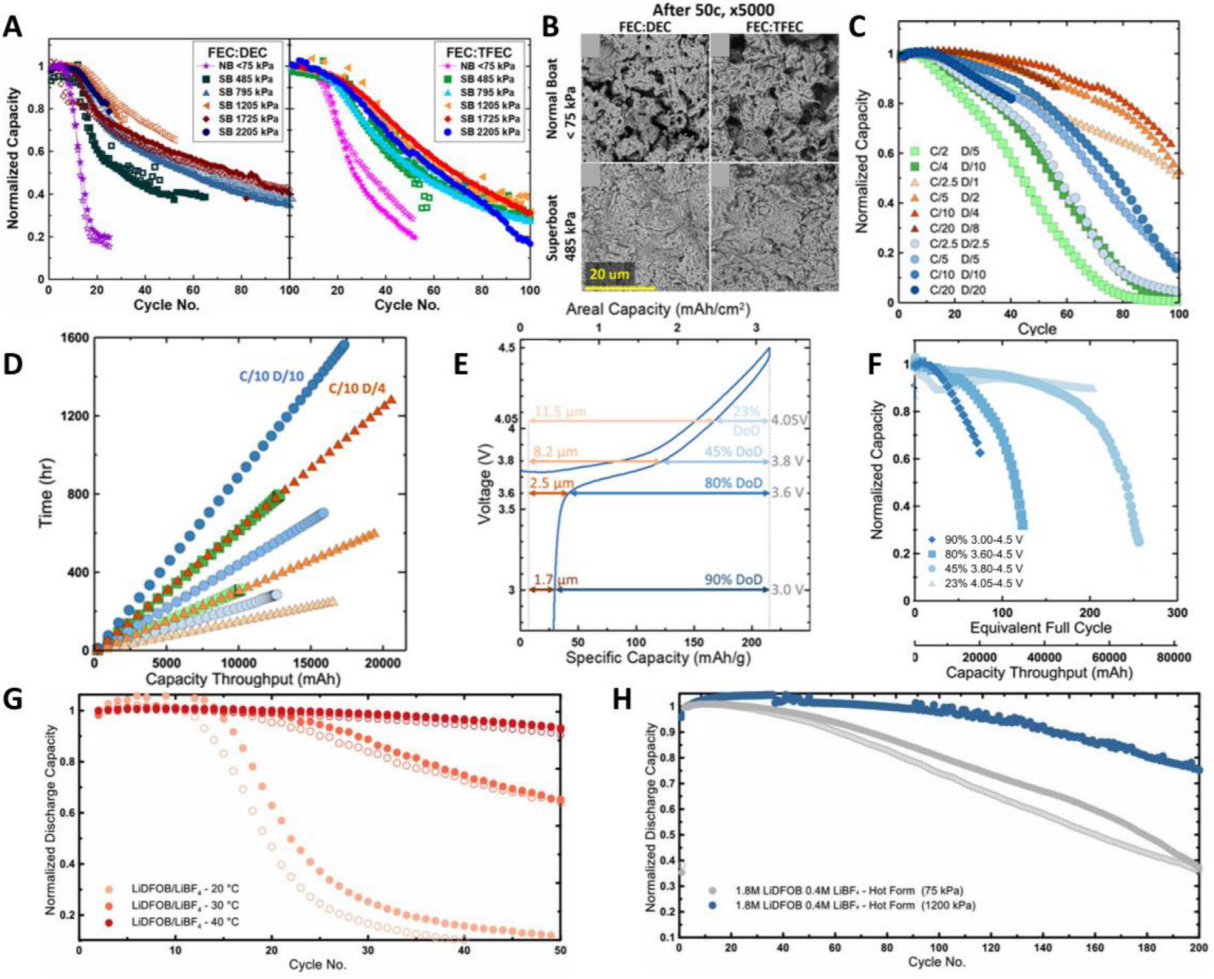
Representative studies on operating protocols for anode‐free lithium metal batteries (AFLMBs). (A,B) Cycle stability of FEC:DEC and FEC:TFEC electrolytes with different external pressure up to 2205 kPa and SEM image of plated Li metal with different electrolytes and pressure after 50 cycles. Reproduced with permission.^[^ ^67^ ^]^ Copyright 2019, The Electrochemical Society. (C–F) Influence of asymmetric fast charge/slow discharge and slow charge/fast discharge on cycle stability, capacity throughput over life time of cells with different current protocols, the theoretical thickness of Li reservoir formed in anode by controlling depth of discharge and normalized capacity throughput with different depth of discharge (DoD). Reproduced with permission.^[^ ^137^ ^]^ Copyright 2021, The Electrochemical Society. (G,H) Cycle stability of LiDFOB/LiBF_4_ electrolytes with different temperatures (20, 30, and 40°C) and outstanding cyclability of concentrated dual salt electrolyte (1.8 m LiDFOB 0.4 m LiBF_4_ FEC/DEC (1:2, v/v)) under pressurized condition. Reproduced with permission.^[^ ^66^ ^]^ Copyright 2019, The Electrochemical Society.

AFLMBs suffer from drastic capacity decay upon cycling.^[^ ^45^, ^68^, ^168^ ^]^ Electrolytes and current collector modifications have improved the CEs of AFLMBs. In addition to the internal components of cells, various operational conditions of cells significantly affect the electrochemical performance.^[^ ^169^, ^170^ ^]^ Among these, the application of external pressure is widely used for (AF)LMBs to reduce SEI cracking and dendrite formation.^[^ ^171^ ^]^ Therefore, most anode‐free pouch cells are tested under pressurized conditions. Dahn et al. investigated the effects of pressurized conditions on anode‐free pouch cells with two different electrolytes (1 m LiPF_6_ dissolved in FEC:DEC and FEC: bis(2,2,2‐trifluoroethyl) carbonate, TEFC).^[^ ^67^ ^]^ Surprisingly, the cyclability of cells containing FEC:DEC was greatly improved to 100 cycles at 1725 kPa in Figure 10A. However, a higher pressure (above 1725 kPa) did not further enhance the cyclability because of electrode degradation by polarization growth. In contrast, the cycle life of cells with FEC:TEFC was saturated at a relatively low pressure of 795 kPa. In addition, as shown in Figure 10B, the morphology of the plated Li at high pressure (485 kPa) with more fluorinated solvents (FEC:TFEC) was more compact than that with FEC:DEC because of the contribution of fluorinated solvents to form LiF‐rich SEI layer. Therefore, although higher pressure is beneficial to Li deposition, proper pressurized conditions should be set considering electrolyte properties.

In addition, the current density seriously affects dendritic growth as Li‐ions are depleted near the surface of Li under a higher current density. In 2020, Louli et al. investigated the effects of symmetric and asymmetric charge/discharge rates.^[^ ^137^ ^]^ They showed that an asymmetric slow charge benefits cyclability more than an asymmetric fast charge or a symmetric charge and discharge (Figure 10C). This was because the slow charge induced a low concentration gradient of Li‐ions near the electrode surface, leading to uniform Li growth. Conversely, fast discharge is beneficial for preferential Li stripping owing to the high localized current density of the protrusions.^[^ ^172^, ^173^ ^]^ Therefore, a flat uniform surface of Li metal was built by an asymmetric faster discharge. However, a slower discharge results in dendritic and tortuous Li metal with high Li‐ion gradients on the electrode surface, which deteriorates the cycle life. As shown in Figure 10D, a high‐capacity throughput was delivered by the asymmetric slower charge.

Also, adjusting the depth of discharge (DoD) affects the cycle stability of the AFLMBs. The Li reservoir could be formed by controlling the discharging cut‐off voltage because Li was not completely removed from the anode owing to the slow kinetics of the cathode during the discharge process (Figure 10E).^[^ ^137^ ^]^ In situ built Li reservoirs are favorable for extending cycle stability until depletion, as in general Li excess LMBs or Li reservoir strategy of ALFMBs. Louli et al. investigated the correlation between the lower cut‐off voltage and the cycle life of anode‐free Cu||NMC532 cells. After fully charged to 4.5 V, various cut‐off voltages were set to investigate the effects of DoD (Figure 10E). As the lower cut‐off voltage is set from 3.0 V (90% of DoD) to 4.05 V (23% of DoD), 1.7–11.5 μm of excess Li reservoir is built. Anode‐free pouch cells with the lowest DoD can maintain capacity without decay for more than 1000 cycles because of the continuous replenishment of Li, while other cells fail more quickly as the DoDs deepen. As shown in Figure 10F, the low DoD protocol is effective in improving cycle stability and capacity throughput. However, since the discharge capacity is very limited, it must be recharged frequently, and practical applications may be difficult. Therefore, it is important to precisely set the cut‐off voltage while carefully considering the balance between cycle stability and discharge capacity. Furthermore, temperature is an important parameter for electrochemistry because it affects the diffusion, viscosity, and rate of the decomposition reaction of the electrolyte.^[^ ^169^, ^174^ ^]^ In particular, Li becomes softer as the temperature increases, inducing lateral and compact morphologies.^[^ ^66^ ^]^ However, decomposition of salts could be accelerated resulting in unsatisfactory cycle performance. Genovese et al. used a hot formation protocol to preserve the electrolyte and benefit from high temperature.^[^ ^66^ ^]^ As shown in Figure 10G, the capacity retention of the anode‐free pouch cell improved when operated at 40°C; in contrast, a large capacity decay occurred for the cells operated at 20°C. However, the cycle stability of the cell operated at low temperature was improved dramatically by two initial cycles at 40°C with asymmetric charge and discharge (C/10 and D/2) between 3.6–4.5 V. This significant improvement in the hot formation protocol comes from the beneficial SEI layer through active CO_2_ gas generation by the decomposition of LiDFOB during hot formation cycles. Combined with high pressure, anode‐free Cu||NMC532 batteries with hot formation protocols operated at low temperatures achieved over 200 cycles with 80% capacity retention (Figure 10H).

## 6 PERSPECTIVE

Despite numerous efforts dedicated to the research of AFLMBs, there are still many challenges to overcome, including their extremely low cyclability. As a result, in this section, we focus on the broad issues and prospects for future research on AFLMBs.

### 6.1 Designing practical electrolytes

Commercial carbonate‐based electrolytes for LIBs are not applicable to practical AFLMBs because of their higher reactivity with Li metal. Even at high operating pressures, the discharge capacity of pouch cells containing commercial carbonate‐based electrolytes reached zero in approximately 30 cycles. In this context, ether‐based electrolytes have attracted considerable attention owing to their compatibility with Li metal. As mentioned earlier, the low oxidation stability, which is considered a major disadvantage of ether‐based electrolytes, was alleviated by HCEs, LHCEs, or fluorination of solvent molecules. In particular, fluorination of solvent molecules not only improves their oxidative stability but also increases the proportion of LiF in the SEI layer, resulting in state‐of‐the‐art anode‐free lithium metal pouch‐cell performance. On the other hand, these strategies have a trade‐off relationship between electrochemical performance and high contents of fluorine elements in electrolytes, which causes severe cost and environmental issues.^[^ ^47^, ^125^ ^]^ As a result, fluorine‐free or low‐fluorine electrolytes have been gaining immense attention in the research field of alkali metal anodes.^[^ ^6^, ^175^ ^]^

### 6.2 Fabricating current collectors for practical application

In Section 2, various fabrication processes have been discussed to enhance the electrochemical reversibility of the Li plating/stripping process on Cu current collectors, including ALD,^[^ ^176^ ^]^ CVD,^[^ ^52^ ^]^ spin coating,^[^ ^50^, ^51^ ^]^ and doctor‐blade coating.^[^ ^105^, ^112^, ^177^ ^]^ However, most papers do not clearly state if these methods are practical for large‐scale production. The current collectors for AFLMBs should be designed to meet the demands of large‐scale production and automatic operation, just like how cathodes and anodes for LIBs are optimized for roll‐to‐roll processes.^[^ ^178^ ^]^ Adopting the roll‐to‐roll process of current LIB production lines to AFLMBs would ensure economic feasibility, which is crucial for commercialization. For example, the silver‐carbon composites,^[^ ^105^ ^]^ which we introduced earlier, can be easily applied to the roll‐to‐roll process, as it is similar to producing graphite anodes for LIBs. Conversely, thin‐film manufacturing processes such as ALD and CVD can be adapted for roll‐to‐roll processes,^[^ ^179^, ^180^ ^]^ but they differ significantly from the current LIB manufacturing processes. Therefore, new production lines are necessary for practical applications. On the other hand, the spin coating technique can only be utilized in batch processes, making it unsuitable for roll‐to‐roll processing. In summary, while there are various approaches to modifying Cu current collectors, only few are applicable to large‐scale production and automated processes. Therefore, in‐depth research on fundamental understandings of current collectors and practical research on achieving high‐energy AFLMBs through modification of current collectors should be carried out simultaneously. Finally, various electrochemical evaluations for the modification of the current collectors should be performed under strict conditions to confirm their feasibility in large‐scale cell.

### 6.3 Practical test conditions

AFLMBs meet the social need for high energy density, which can maximize the range of electric vehicles and expand their applications. Accordingly, a low E/C ratio (amount of electrolyte to the capacity of the cathode, under 3 g Ah^−1^) and high areal capacity of the cathode (over 3.8 mA h cm^−2^) should be satisfied to achieve a high energy density AFLMBs.^[^ ^181^ ^]^ However, many papers have recently reported the fabrication of anode‐free lithium metal full cells with low‐loading cathodes and flooded electrolytes. In such cases, assessments of the practical application of the strategy may be doubted because the cyclability of AFLMBs under harsh conditions is completely different from that under mild conditions. For instance, when 4 mA h cm^−2^ of the cathode is operated at 0.25 C, a current density of 1 mA cm^−2^ is applied to the anode (if the sizes of the cathode and anode are equal), but in the case of 1 mA h cm^−2^ an equal current density is applied at 1 C. In addition, electrolyte drying due to the reaction with Li metal increases dramatically with Li utilization. Consequently, the test protocol and conditions should be carefully established to demonstrate the practical application of this strategy.

### 6.4 Introducing sacrificial cathode additives

The inherent limitations of the AFLMBs are obvious. With zero excess Li, even if a CE of 99.9% is achieved, only 80% of the initial capacity can be maintained for 223 cycles (Table 1). Therefore, sacrificial cathode additives, such as Li_2_O^[^ ^64^ ^]^ and Lithium oxalate (LO)^[^ ^65^ ^]^ which provide excess Li at the cathode, should be introduced to extend the cycle life of AFLMBs. Because an LMB with a thin Li anode suffers from (1) high production cost, (2) energy‐intensive, repetitive rolling process, and (3) thickness control limit,^[^ ^182^, ^183^ ^]^ sacrificial cathode additive in AFLMBs is considered to be a better option for excess Li. Moreover, the introduction of sacrificial additives slightly reduces the energy compared to the typical AFLMBs, but it is believed that a high energy density and cyclability can be achieved simultaneously by investigating the optimal point for the capacity of the sacrificial additive. Finally, it is anticipated that by simultaneously implementing the various fundamental approaches outlined above, many issues with AFLMBs can be resolved.

## 7 CONCLUSION

In conclusion, AFLMBs are regarded as promising systems because of their unique cell configuration, in which the energy density is maximized. Furthermore, the absence of highly reactive Li metal for initial cell fabrication gives rise to significant advantages, such as easy processability, reduced cost, and high safety. However, the rapid capacity degradation and low cyclability by parasitic reaction of Li and low reversibility are major concerns for AFLMBs. In this review, fundamental issues and major challenges are presented, and recently reported major strategies are summarized. To overcome the challenging nature of metallic Li, many efforts have been devoted to suppressing dendrites, dead Li, and electrolyte consumption by focusing on developing beneficial layers through the artificial coating on current collectors and electrolyte modification. In addition, cathode engineering and various protocols are proven to be effective in extending the cycle life of AFLMBs. To cope with the limitation of the limited inventory of Li, these strategies should be combined to give a synergistic effect in enhancing the reversible reaction of Li. Finally, as we provide in perspective, practical electrolytes and current collector modifications should be considered and tested with practical testing conditions and the impact of various sacrificial agents should be explored for future AFLMBs.

## AUTHOR CONTRIBUTIONS

Cheol‐Young Park and Jinuk Kim contributed equally to this work. Won‐Gwang Lim and Jinwoo Lee supervised the manuscript.

## CONFLICTS OF INTEREST STATEMENT

The authors declare no conflicts of interest.
